# Integrative genomic analyses for identification and prioritization of long non-coding RNAs associated with autism

**DOI:** 10.1371/journal.pone.0178532

**Published:** 2017-05-31

**Authors:** Brian L. Gudenas, Anand K. Srivastava, Liangjiang Wang

**Affiliations:** 1 Department of Genetics and Biochemistry, Clemson University, Clemson, South Carolina, United States of America; 2 J.C. Self Research Institute of Human Genetics, Greenwood Genetic Center, Greenwood, South Carolina, United States of America; National Center for Biotechnology Information, UNITED STATES

## Abstract

Genetic studies have identified many risk loci for autism spectrum disorder (ASD) although causal factors in the majority of cases are still unknown. Currently, known ASD risk genes are all protein-coding genes; however, the vast majority of transcripts in humans are non-coding RNAs (ncRNAs) which do not encode proteins. Recently, long non-coding RNAs (lncRNAs) were shown to be highly expressed in the human brain and crucial for normal brain development. We have constructed a computational pipeline for the integration of various genomic datasets to identify lncRNAs associated with ASD. This pipeline utilizes differential gene expression patterns in affected tissues in conjunction with gene co-expression networks in tissue-matched non-affected samples. We analyzed RNA-seq data from the cortical brain tissues from ASD cases and controls to identify lncRNAs differentially expressed in ASD. We derived a gene co-expression network from an independent human brain developmental transcriptome and detected a convergence of the differentially expressed lncRNAs and known ASD risk genes into specific co-expression modules. Co-expression network analysis facilitates the discovery of associations between previously uncharacterized lncRNAs with known ASD risk genes, affected molecular pathways and at-risk developmental time points. In addition, we show that some of these lncRNAs have a high degree of overlap with major CNVs detected in ASD genetic studies. By utilizing this integrative approach comprised of differential expression analysis in affected tissues and connectivity metrics from a developmental co-expression network, we have prioritized a set of candidate ASD-associated lncRNAs. The identification of lncRNAs as novel ASD susceptibility genes could help explain the genetic pathogenesis of ASD.

## Introduction

Autism spectrum disorder (ASD) is a group of highly heritable genetic neurodevelopmental disorders characterized by impaired social communications with an estimated prevalence of 1 out of 68 births in 2010 [1]. ASD risk genes include hundreds of protein-coding genes most commonly affected by copy number variants (CNVs) which can perturb gene expression; however, each known ASD risk gene only accounts for less than a few percent of ASD cases [2,3]. Most of these ASD risk genes function in several biological pathways, such as synaptic transmission, transcriptional regulation, immune response and chromatin remodeling [4–7]. ASD is highly heterogeneous and there are still unaccounted genetic risk factors that likely reside outside protein-coding regions such as in regulatory non-coding RNAs (ncRNAs).

Long non-coding RNAs (lncRNAs) are transcripts greater in length than 200 nucleotides, which do not encode proteins [8]. LncRNAs have been shown to be involved in a diverse array of neurodevelopmental functions such as brain development, neural differentiation and synaptic plasticity [9–12]. LncRNAs can also have epigenetic functions by interacting with chromatin re-modeling complexes to facilitate gene silencing or activation [10,13]. In addition, lncRNAs function in brain development, contributing to increased cognitive function and neuronal tissue specification [14]. Other than epigenetic functions, studies have shown lncRNA regulatory actions through diverse mechanisms such as multi-protein scaffolding, transcriptional interference, post-transcriptional modification and miRNA blocking [15]. The functionalities of lncRNAs to negatively or positively affect gene expression at the transcriptional, post-transcriptional, translational and epigenetic levels exhibit their regulatory versatility. In addition, because lncRNAs are highly tissue-specific and expressed at relatively high levels in the human brain, they are likely involved in complex neurodevelopmental disorders such as ASD [9,14,16–18].

Microarray gene expression profiling of the ASD cortex indicated that the number of differentially expressed lncRNAs between the prefrontal cortex and cerebellum in ASD brains was lower than between the same brain regions in controls [17]. This paradigm has also been observed in other studies in the ASD cortex with regards to mRNA expression and differentially methylated regions [19,20]. These studies suggest that ASD may be caused by aberrant neurodevelopment which could dysregulate neuronal tissue-specification. In a differential expression analysis of ASD leukocytes, more lncRNAs were found to be differentially expressed than mRNAs, including thirteen lncRNAs associated with synaptic functions [16]. Genetic lesions of these lncRNAs, such as by CNVs, may impair gene expression and/or regulation which could have downstream regulatory consequences affecting neurodevelopment [21–24].

Our goal is to find differentially expressed lncRNAs in the ASD cortex and then identify which lncRNAs are also highly co-expressed with known ASD risk genes and ASD-affected biological pathways in neurodevelopment. The reasoning for using co-expression networks is that lncRNAs are vastly functionally uncharacterized, therefore, highly correlated gene and lncRNA expression patterns across developmental time imply shared biological function and/or regulation. Co-expression allows us to refine our list of candidate ASD-associated lncRNAs through gained functional insights from this expression-based guilt-by-association analysis. We have utilized an integrative approach for identifying ASD-associated lncRNAs, by analyzing differentially expressed lncRNAs in the ASD cortex and mapping them onto a brain developmental gene co-expression network. This approach, despite the genetic heterogeneity of ASD, facilitates the identification of ASD-associated lncRNAs by leveraging the information of ASD and non-ASD developmental cortex transcriptomes.

## Results and discussion

### Differential expression of lncRNAs in the ASD cortex

**Fig 1:**
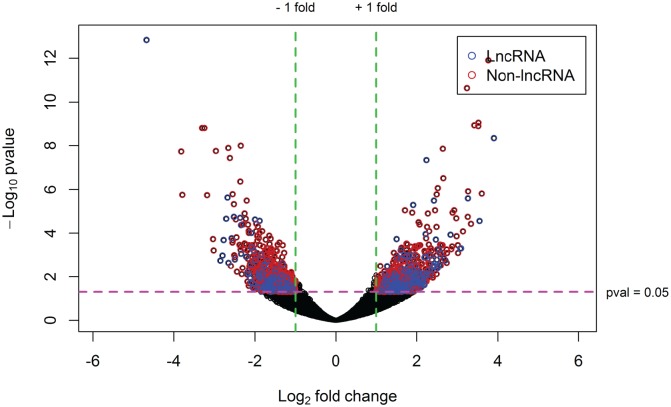
Differentially expressed genes in the ASD cortex. The volcano plot displays genes differentially expressed in the ASD cortex. A gene was required to have an absolute value of log_2_ fold change greater than or equal to one and an adjusted p-value less than 0.05 to be considered differentially expressed.

We speculated that genes differentially expressed in the ASD cortex would be informative for identifying ASD-associated lncRNAs because the human cortex has been implicated in ASD pathophysiology by multiple transcriptomics studies [25,26]. Therefore, we re-analyzed RNA-seq data from the ASD cortex from a previous independent study which focused on differential splicing of protein-coding genes, yet did not analyze lncRNAs [20]. We found 1602 differentially expressed genes (FDR adjusted p-value < 0.05; |Log_2_ fold change| ≥ 1) (**Fig 1**). Furthermore, genes significantly down-regulated in the ASD cortex were enriched for biological processes related to synaptic function such as chemical synaptic transmission and synaptic signaling (p-values < .001) (**S1 Text**). The up-regulated genes were enriched for biological functions such as immune system process, cell surface receptor signaling pathway and response to cytokines (p-values < .001) **(S1 Text)**. These results are in concordance with previous findings from ASD brain gene expression studies; genes functioning in the synaptic transmission pathway were down-regulated while genes involved in immune response were upregulated [5]. Furthermore, known ASD risk genes, curated by the Simons Foundation Autism Research Initiative (SFARI), were enriched within the differentially expressed genes (p-value < .001) (**S1 Text**) [27]. Thus, differentially expressed genes in the ASD cortex appear to be representative of known ASD pathophysiology based on the enrichment of biological pathways dysregulated in ASD and the overrepresentation of known ASD risk genes.

Interestingly, we detected 263 lncRNAs differentially expressed between ASD and control cortical brain samples (FDR adjusted p-value < 0.05; |Log_2_ fold change| ≥ 1) (**S1 Table**). Almost half of these differentially expressed lncRNAs were from intergenic regions (45%), with most of the remaining lncRNAs antisense to protein-coding genes (41%) (**S2 Text**). Next, we identified the nearest neighboring gene to each lncRNA since lncRNAs can have cis-regulatory mechanisms; remarkably, 5 of these lncRNAs are antisense to known ASD risk genes, including RAPGEF4, DLX6, STXBP5, KLC2 and DMXL2 (**S2 Table**).

Next, we asked if these lncRNAs are selectively expressed in the brain relative to the other tissue types, which would suggest brain-specific functions. We extracted RNA-seq data from the Genotype-Tissue Expression project for over 40 different human tissues, each containing over 50 samples, and plotted the median expression of the differentially expressed lncRNAs for each tissue [28] (**S1 Fig**). The majority of these lncRNAs are highly expressed in brain tissues relative to the other tissue types (**S1 Fig**). Furthermore, we found that the tissue type with the highest average expression for these lncRNAs is the brain cortex, suggesting that these lncRNAs perform cortical-associated biological functions (**S1 Fig**). We now have a list of lncRNAs differentially expressed in the ASD cortex, and approximately 50% of these lncRNAs have a fractional expression level greater than 50% in the human brain, suggesting tissue selectivity (**S2 Table**). We further refine the list of candidate lncRNAs through co-expression network analysis.

### Gene co-expression network analysis indicates that differentially expressed lncRNAs are involved in biological processes dysregulated in ASD

We built genome-wide gene co-expression networks by utilizing the BrainSpan developmental transcriptome dataset, which consists of brain samples from eight weeks post-conception up to 40 years of age [29]. First, we extracted all samples within cortical brain regions and then filtered out lowly expressed genes. This pre-processing resulted in a final RNA-seq dataset consisting of 352 cortical brain samples and 26,188 genes, of which 127 out of the 263 differentially expressed lncRNAs were present. Next, we used this refined RNA-seq dataset for signed weighted gene co-expression network analysis (WGCNA) [30], and identified 33 gene co-expression modules (**S1 Table**). These co-expression modules symbolize groups of genes with similar expression patterns through cortical development. Measuring the co-expression of randomly sampled groups of genes of equal size to each module shows that these co-expression modules are statistically significant (**S2 Fig**).

**Fig 2:**
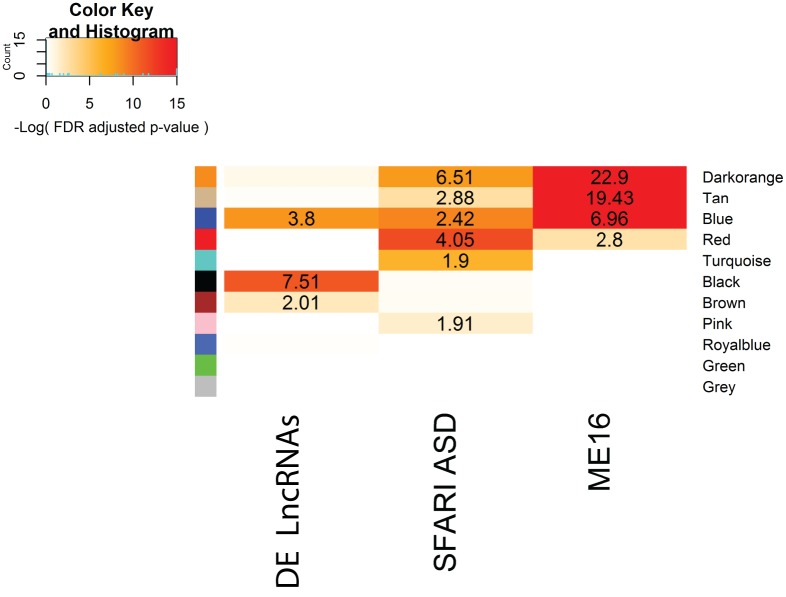
Enrichment of lncRNAs and ASD genes in brain developmental co-expression modules. The heatmap shows module-based enrichment of gene lists. “DE LncRNAs” are lncRNAs differentially expressed in the ASD cortex, “SFARI ASD” are known ASD risk genes, and “ME16” is an ASD-associated gene co-expression module identified in an independent study. Enrichment of gene lists was determined by a Fischer’s exact test requiring the FDR-adjusted p-value < 0.05 and an Odds Ratio > 1. Only modules containing at least 1 differentially expressed lncRNAs are shown.

Next, we asked if there were modules enriched for both differentially expressed lncRNAs and known ASD risk genes. To assess the enrichment of ASD risk genes within modules, we utilized two independent lists of ASD risk genes. The list referred to as SFARI has been curated by the Simons Foundation Autism Research Initiative (SFARI) and these genes are scored based on the degree and strength of evidence for implications in ASD [27]. To avoid any bias in the SFARI gene set, which was manually curated, we also utilized a gene list known as the ME16 module which was identified in an independent unsupervised genome-wide co-expression study in brain tissue [26]. The ME16 gene list was shown to be enriched for genes with rare *de novo* genetic variants in ASD probands and a gene list known as “asdM12”, which contains genes aberrantly expressed in the ASD cortex [20,26]. The differentially expressed lncRNAs show statistical enrichment in the Blue, Brown and Black modules (**Fig 2**). Interestingly, the Blue module is also enriched for SFARI ASD risk genes and ME16 genes (**Fig 2**). The co-enrichment of two ASD gene sets and lncRNAs differentially expressed in the ASD cortex within the same developmental brain co-expression module suggests that the Blue module and the lncRNAs within it may be functionally involved in ASD pathogenesis.

**Fig 3:**
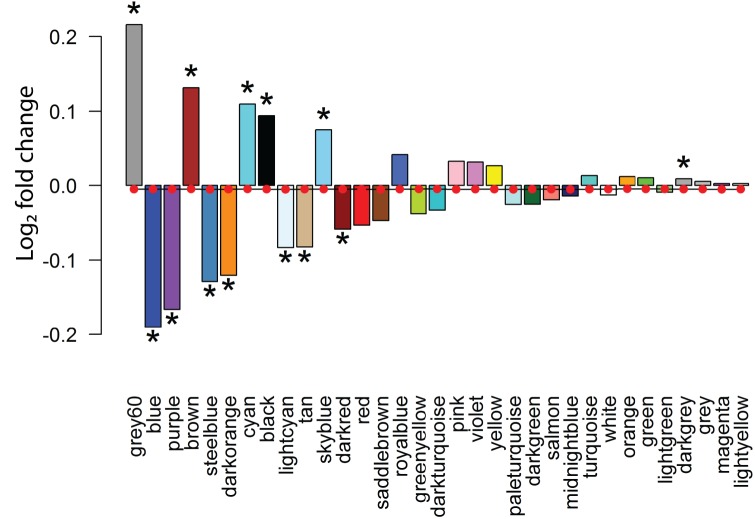
Differential expression in the ASD cortex overlaid onto developmental co-expression modules. The average log_2_ fold changes of genes differentially expressed in the ASD cortex were overlaid onto the co-expression modules formed using the BrainSpan Developmental Transcriptome. Any genes that failed to reach significance had their log_2_ fold changes set to 0. The red circle within each bar plot is the average log_2_ fold change of 10,000 random gene samplings of equal size to the respective module. Significance of differential expression compared to the permuted distribution (FDR-adjusted p-value < 0.05) is denoted by a black asterisk adjacent to a modules respective bar plot.

Next, we asked if the identified co-expression modules were dysregulated in ASD by assessing their average differential expression in the ASD cortex for each module. Since we have identified less than two thousand differentially expressed genes in the ASD cortex and there are over 26,000 genes in our developmental co-expression network, the vast majority of genes in the network have an ASD cortical fold change of zero (log_2_ fold change). Therefore, modules showing a statistically significant average ASD fold change which deviates from zero likely represent biological pathways dysregulated in ASD. When overlaying the differential expression fold changes calculated from the ASD cortex onto the co-expression modules, 13 out of the 33 modules were found to be significantly differentially expressed when compared to randomly sampled gene sets of the same size (**Fig 3**). Interestingly, all modules enriched for lncRNAs are differentially expressed in ASD; the Blue module shows down-regulation while the Brown and Black modules are up-regulated (**Fig 3**). This suggests common mechanisms dysregulating these ASD-associated gene networks. Next, we examine the functional enrichment of these ASD-associated modules.

**Fig 4:**
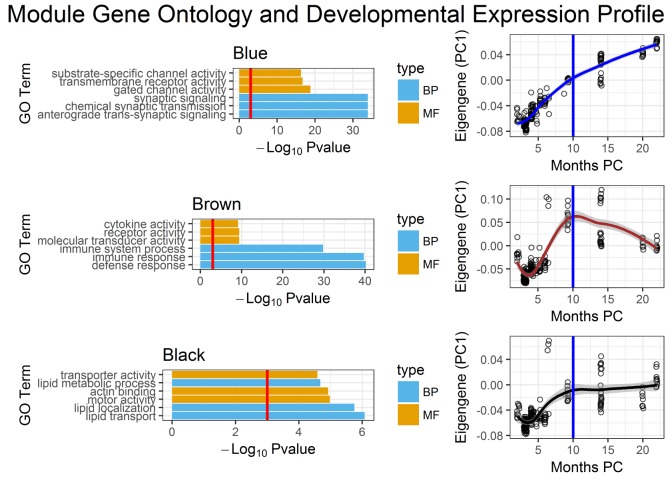
Characterization of modules enriched for differentially expressed lncRNAs. Gene Ontology functional enrichment was analyzed for each module and adjusted for multiple comparisons (FDR < 0.05). The scatterplots show modular developmental expression profiles based on a module eigengene (1^st^ principal component) through developmental time, months PC means months post-conception (2 months post-conception to 1 post-natal year), with the blue vertical line demarcating birth. The trend line of each scatterplot is derived from a locally weighted scatterplot smoothing function.

We functionally characterized all modules enriched for differentially expressed lncRNAs by performing Gene Ontology enrichment analysis and by visualizing their developmental expression pattern in the human cortex (**Fig 4**). The Blue module’s top three enriched biological processes are synaptic signaling, chemical synaptic transmission and anterograde trans-synaptic signaling (p-values < .001) (**Fig 4**). The synaptic transmission pathway is a well-known biological process dysregulated in ASD from gene expression and genome-wide association studies [3,4,7,17,23,24,27,31,32]. Moreover, the expression of the genes within the Blue module shows a positive correlation with developmental time in the cortex (Pearson’s correlation coefficient = 0.55) (**Fig 4**), possibly coinciding with major cortical development [26]. The Brown and Black modules, which are only enriched for differentially expressed lncRNAs, have the functional enrichment for immune response and lipid transport, respectively (**Fig 4**). Perturbation in the immune system as well as the transport of fatty acids has also been associated with ASD [33,34]. Remarkably, the differentially expressed lncRNAs are enriched in modules which have been functionally linked with ASD, the synaptic transmission, immune response and lipid transport pathways [4–6,33,35].

### Prioritization of candidate ASD-associated lncRNAs

The Gene Ontology enrichments and developmental expression trajectories (**Fig 4**) are representative of entire co-expression modules, however the differentially expressed lncRNAs represent a minority of the total genes within the modules. To assess the direct relationships between lncRNAs and ASD risk genes, we examined the co-expression solely between lncRNAs and ASD risk genes compared to random permutations. In both ASD gene sets, SFARI and ME16, we find statistically significant (p-values < .0001) summed correlations directly between the differentially expressed lncRNAs and ASD gene sets relative to randomly sampled gene sets (**S3 Fig**). The results further suggest that the identified lncRNAs are involved in the similar convergent biological processes dysregulated in ASD.

**Fig 5:**
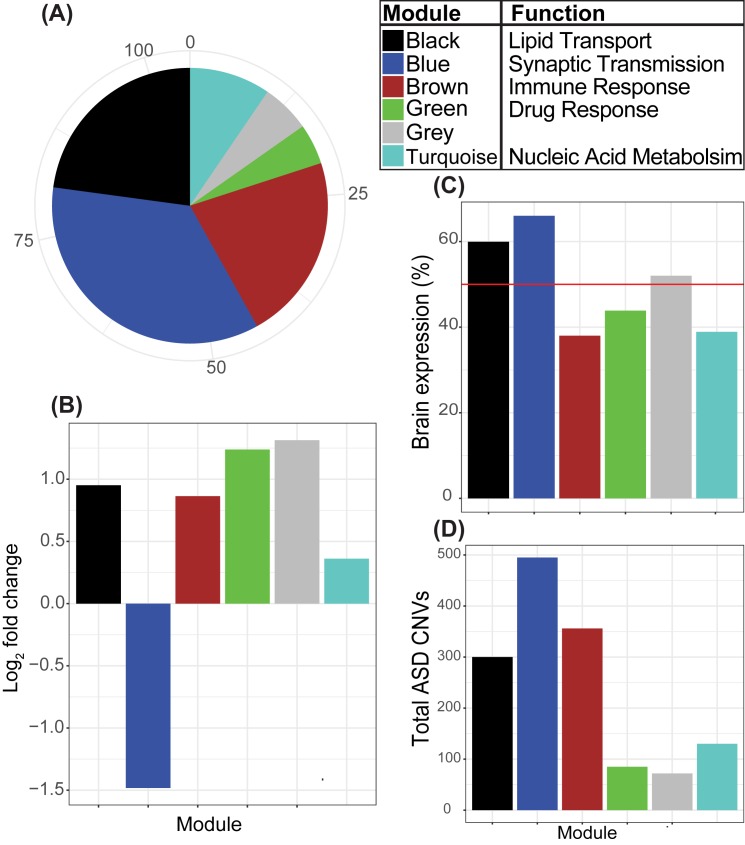
Candidate ASD-associated lncRNA characteristics. (A) Module assignment for differentially expressed lncRNAs. Only modules with at least 4 lncRNAs are displayed. In addition, we provide the module function, which is the highest scoring GO biological process for the whole module. (B) Average log_2_ fold change of expression in the ASD cortex for the differentially expressed lncRNAs from each module. (C) Average fractional expression levels in the brain for the differentially expressed lncRNAs from each module. Fractional brain expression for each lncRNA is calculated using the RNA-seq data from the Genotype-Tissue Expression project as the total expression in brain tissues divided by the sum of expression across all tissue types [28]. The red line at 50% represents the threshold for tissue specificity as defined by Ayupe et al [36]. (D) Overlaps between ASD CNVs and DE lncRNAs from each module.

All the evidence presented thus far has been expression-based; therefore, we speculated that the integration of genetic mutational data, such as ASD-associated CNVs would be beneficial for the ranking of candidate ASD-associated lncRNAs. We incorporated a list of 5,030 major ASD-associated CNVs curated by SFARI from ASD genetic studies and calculated overlaps for the genes in the developmental co-expression network. To prioritize differentially expressed lncRNAs, we ranked the lncRNAs based on their module assignment to prioritize lncRNAs in modules enriched for ASD risk genes followed by ranking lncRNAs within the same module by their total overlaps with ASD-associated CNVs. Interestingly, the highest ranked lncRNA, HTR5A-AS1, is highly brain-specific and its most highly correlated gene in the network is AGBL4, a known ASD risk gene which is also down-regulated in the ASD cortex (Pearson correlation = 0.98) (**S2 Table**). The genomic characteristics and co-expression network results for the candidate ASD-associated lncRNAs, grouped by module, are presented in **Fig 5**. In summary, we observe the largest amount of the differentially expressed lncRNAs in the Blue, Black and Brown modules (**Fig 5A**); the lncRNAs in the Blue module are down-regulated in the ASD cortex while the lncRNAs in the Black and Brown modules are up-regulated (**Fig 5B**). Furthermore, the lncRNAs in the Blue and Black modules exhibit tissue selectivity to the brain relative to all other tissue types (**Fig 5C**). In addition, the lncRNAs of the Blue module show the highest level of ASD CNV overlaps (**Fig 5D**), suggesting that these lncRNAs may be affected by CNVs in ASD.

For each differentially expressed lncRNA, we also identified the most highly co-expressed protein-coding gene within the developmental co-expression network. Remarkably, fourteen of these most highly co-expressed genes are known ASD risk genes (**S2 Table**). This result indicates that these lncRNAs are likely involved in the molecular function and/or regulation of the specific ASD risk genes. However, further experimental studies will be needed to decipher the true relationship between these candidate ASD-associated lncRNAs and their highly co-expressed ASD risk genes. This prioritized list of ASD-associated lncRNAs can assist geneticists by providing high-quality novel experimental targets to further elucidate ASD pathogenesis.

## Conclusions

Utilizing differential expression analysis in affected tissues coupled with an independent developmental gene co-expression network, we have identified a list of candidate ASD-associated lncRNAs. These lncRNAs are differentially expressed in the ASD cortex, highly expressed in brain tissues and also co-expressed with ASD risk genes in the developing cortex. We have identified a co-expression module enriched for both differentially expressed lncRNAs and ASD risk genes; this module is functionally enriched for the synaptic signaling and transmission pathways. In addition, two modules are enriched solely for differentially expressed lncRNAs, which are functionally enriched for the immune response and lipid transport pathways, biological processes suspected to be dysregulated in ASD. Furthermore, the modules enriched for lncRNAs are differentially expressed in the ASD cortex, with the synaptic module showing down-regulation and the immune and lipid transport modules being up-regulated. Finally, we have identified 14 lncRNAs whose most highly co-expressed genes in the entire network are known ASD risk genes, suggesting direct functional associations. The convergence of the independent genomic results presented in this study suggests an association between the previously uncharacterized lncRNAs and ASD in the human brain. These lncRNAs can serve as prioritized candidate ASD risk genes to expedite future genetic studies on ASD pathogenesis.

## Methods

### RNA-seq data analysis

We extracted raw RNA-sequencing data from human cortical tissues for three ASD cases and controls, each with two technical replicates, from a published study (GEO accession GSE30573) [20]. Transcript abundance was quantified from RNA-seq reads which were mapped to the human transcriptome version GRCh38 with gene annotations from ENSEMBL v86, using Salmon (v0.7.1)[37]. Transcript counts were then imported into R (version 3.3.2) using tximport (R package, v1.2.0)[38]. All subsequent analyses were performed in R (version 3.3.2) on a 64-bit Windows 7 system.

### Differential expression analysis

Differential expression analysis was performed based on the difference in gene counts between ASD cases and controls using DESeq2 (R package, v1.10.1) [39]. DESeq2 estimates differential expression using a negative binomial model which have been shown to be reduce false positives compared to other methods [40]. A gene was considered differentially expressed if it had an FDR-adjusted p-value less than 0.05 and an absolute log_2_ fold change greater than or equal to one.

### Tissue-specific expression of lncRNAs

We downloaded the tissue-specific RNA-seq dataset, in which gene-level median RPKM values are reported for each tissue type, from the Genotype-Tissue Expression (GTEx) project [28]. We filtered the data to remove any tissue types with less than 50 samples. For each differentially expressed lncRNA, FPKM values were Z-score normalized across the tissues then plotted in a heatmap using the gplots (R package, v3.0.1) [41]. Hierarchical clustering was performed on the tissues, and all brain tissues were highlighted blue using a color key. Fractional brain expression is calculated by the summation of the expression values in brain tissues divided by the total expression for all tissue types multiplied by 100 to get the relative percentage.

### Gene lists

The “DE lncRNA” gene list is composed of all significant differentially expressed genes which have lncRNA biotypes according to Ensembl (v84). The ASD gene list was extracted from AutDB, the SFARI human gene database [27]. We utilized the SFARI “Gene Score”, which categorizes ASD risk genes based on evidence for implication in ASD, to filter the ASD risk genes. The “SFARI” gene list is only composed of genes with evidence levels 1–5 (high evidence–minimal evidence). We obtained M16 from an independent genomics study which applied WGCNA to the BrainSpan developmental RNA-seq dataset to identify modules in the developing brain enriched for ASD risk genes [26].

### Cortical gene expression data

We downloaded the BrainSpan developmental transcriptome Gencode v10 dataset (summarized to gene-level reads) from http://www.brainspan.org/. Only samples from cortical brain regions were used in our analysis and we used a variance filter to remove the bottom two quartiles of genes based on across-sample variance. This preprocessing resulted in an RNA-seq dataset with 352 cortical samples and 26,188 genes, which was used as input for gene co-expression network analysis.

### Co-expression network analysis

Co-expression networks were built using WGCNA (R package, v1.51) [30]. The biweight midcorrelation, a correlation metric more robust to outliers than the Pearson correlation, was used to calculate correlations between all gene pairs. Afterwards, a signed weighted network was created using a soft-threshold power of 12 to approximate network scale-free topology. Next, the topological overlap was calculated between all gene pairs. The topological overlap metric represents not only pair-wise relationships, but relationships between cliques of genes and is therefore advantageous for clustering genes over pair-wise methods. To identify co-expression modules, a hierarchical cluster tree is created based on the topological overlap matrix, with modules representing distinct branches of the dendrogram. Modules are formed from the result of a dynamic tree cutting algorithm [30]. All parameters used for network construction are included in (**S2 Text**).

### Network validation

To validate that a module was co-expressed above what would be expected by random chance, we performed a co-expression permutation test. This was done by assessing the average correlation of randomly sampled gene sets, which were equal in size to the modules derived previously, 10,000 times. Comparing the distributions of the average biweight mid-correlation of randomly sampled gene sets with the co-expression of each module, we found all modules to be significantly co-expressed above random chance (p < 1x10^-4^).

Next, we asked if the up- and down-regulated genes identified in the ASD brain segregated into the developmental modules created using BrainSpan [29]. We performed another permutation test in which we calculated the average log_2_ fold change of 10,000 randomly created gene sets equal in size to each module and then compared these distributions to each module’s average log_2_ fold change in the ASD brain.

### Gene set enrichment analysis

Overrepresentation of gene lists within modules was analyzed using a one-sided Fisher exact test to assess the statistical significance. All p-values, from all gene sets and modules, were adjusted for multiple testing using the False Discovery Rate method [42]. We required an odds ratio > 1 and an adjusted p-value < 0.05 to claim that a gene set is enriched within a module. The–log10 (p-values) was then plotted in a heatmap using the gplots (R package, v3.0.1) [41].

### Module characterization

Gene Ontology enrichment analysis in each lncRNA-enriched module was performed using the GOstats (R Package, v2.36), reported biological processes were required to have an FDR-adjusted p-value < 0.05 [43]. Module eigengenes, representing module developmental trajectories, from 8 weeks post-conception to 1 post-natal year were plotted and fitted with a locally weighted scatterplot smoothing function using ggplot2 (R package, v2.1.0) [44].

### LncRNA-ASD risk gene co-expression analysis

The summation of all biweight midcorrelations between differentially expressed lncRNAs with SFARI or ME16 genes was calculated. Next, the summed correlation between lncRNAs and randomly sampled gene sets of equal size to the respective ASD gene list was calculated 10,000 times. This resulted in a permuted normal distribution, from which a p-value was derived for the actual summed correlation between the lncRNAs and ASD gene sets.

### CNV analysis

CNV summary data were downloaded from SFARI and filtered to only retain CNVs with a report class of “Major” [45]. Using the cytoband locus we converted all CNVs into hg38 genomic coordinates according to the hg38 cytoband coordinates from UCSC (**S2 Text**). Overlaps were quantified for each gene if its genomic coordinates overlapped the CNV range using the GenomicRanges (R package, v1.22.4) [46].

## Supporting information

S1 Fig Tissue specificity of differentially expressed lncRNAs. The heatmap displays lncRNAs median FPKM abundance for each human tissue type. LncRNAs were Z-score normalized across tissue types, and then hierarchical clustering was performed on the tissue types. The blue boxes adjacent to the hierarchical cluster tree denote if the tissue type is derived from the brain.
(TIF)

S2 Fig Co-expression of developmental gene modules. The bar plot shows the average biweight midcorrelation of all gene modules in the brain developmental network. The red circle within each bar represents the average biweight midcorrelation of 10,000 randomly selected gene sets of equal size to the genes within the respective module.
(TIF)

S3 Fig Co-expression of ASD gene sets with differentially expressed lncRNAs. The histograms display the summed biweight midcorrelation between ASD gene sets and 10,000 randomly selected gene sets of equal size to the differentially expressed lncRNAs. The red vertical line represents the sum of the biweight midcorrelation between an ASD gene set with the differentially expressed lncRNAs. P-values were calculated based on the difference between the actual summed correlation (red line) and the permuted normal distribution and adjusted for multiple comparisons (adjusted p-values < 0.001).
(TIF)

S1 Text Characteristics of genes differentially expressed in ASD. The information includes Gene Ontology functional enrichment of up- and down-regulated genes in the ASD cortex, and enrichment of ASD risk genes in the significantly differentially expressed genes of the ASD cortex.
(DOCX)

S2 Text R markdown document containing source code and exploratory data analysis. (DOCX)

S1 Table Weighted gene co-expression network analysis results. The table contains the ASD log_2_ fold change values and resulting q-values for all genes in the network along with the WGCNA module assignment scores.
(CSV)

S2 Table Prioritized ASD-associated lncRNAs. The table contains prioritized candidate ASD-associated lncRNAs, ranked by module. Modules enriched for ASD risk genes are prioritized, followed by modules enriched for lncRNAs. LncRNAs within the same module are ranked in descending order of overlaps with ASD-associated CNVs. For each lncRNA, we also show the log_2_ fold change in the ASD cortex, module assignment within the developmental co-expression network, the most highly correlated (Cor) protein-coding gene in the network, and the SFARI ASD risk score for each correlated gene.
(XLSX)
