# Supplementary figures and images for: Integrative genomic analyses for identification and prioritization of long non-coding RNAs associated with autism
Source: PLoS One. 2017 May 31;12(5):e0178532. doi: 10.1371/journal.pone.0178532 (PMC5451068; doi:10.1371/journal.pone.0178532)

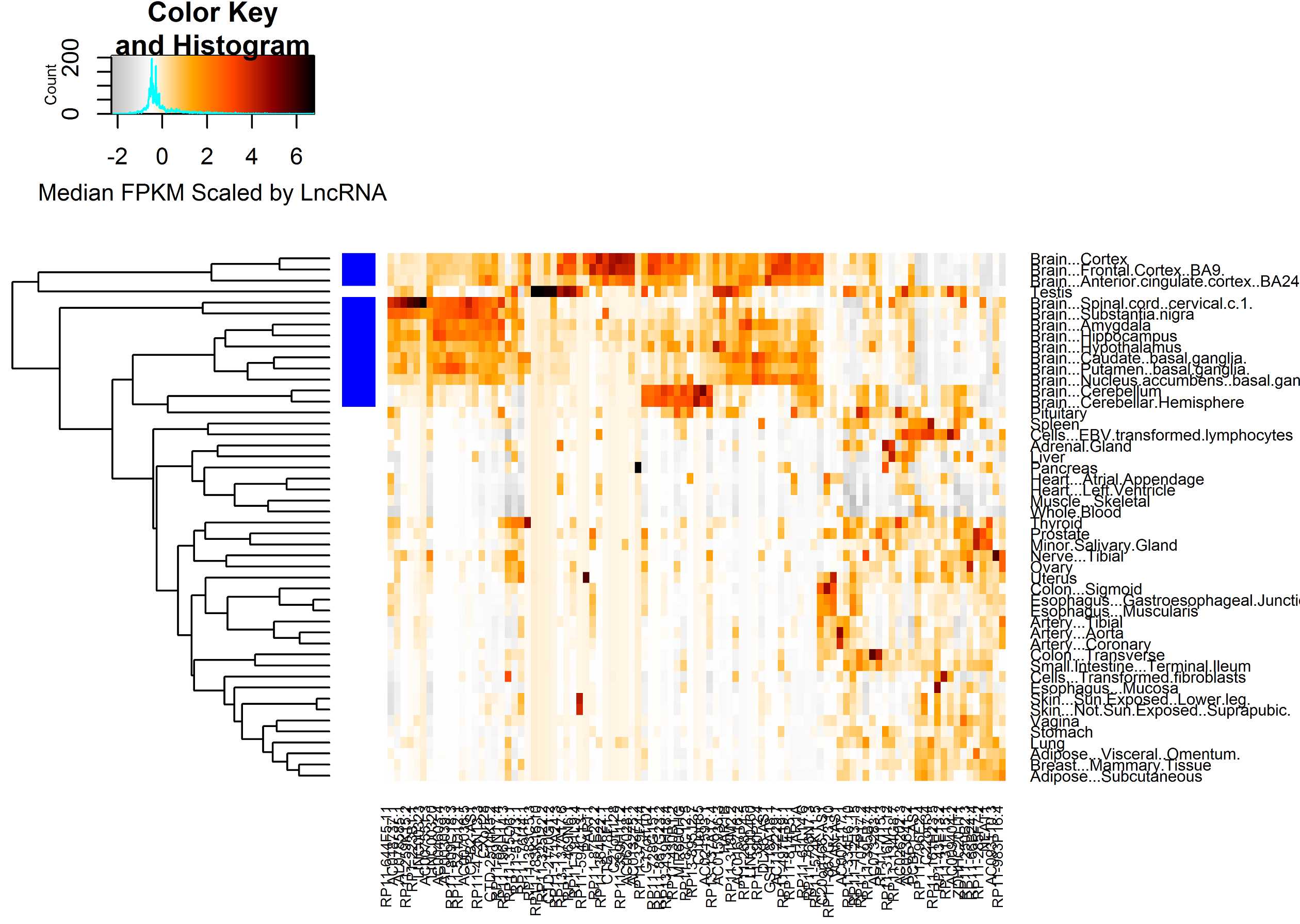

Supplement: S1 Fig — The heatmap displays lncRNAs median FPKM abundance for each human tissue type. LncRNAs were Z-score normalized across tissue types, and then hierarchical clustering was performed on the tissue types. The blue boxes adjacent to the hierarchical cluster tree denote if the tissue type is derived from the brain. (TIF) [file pone.0178532.s001.tif]

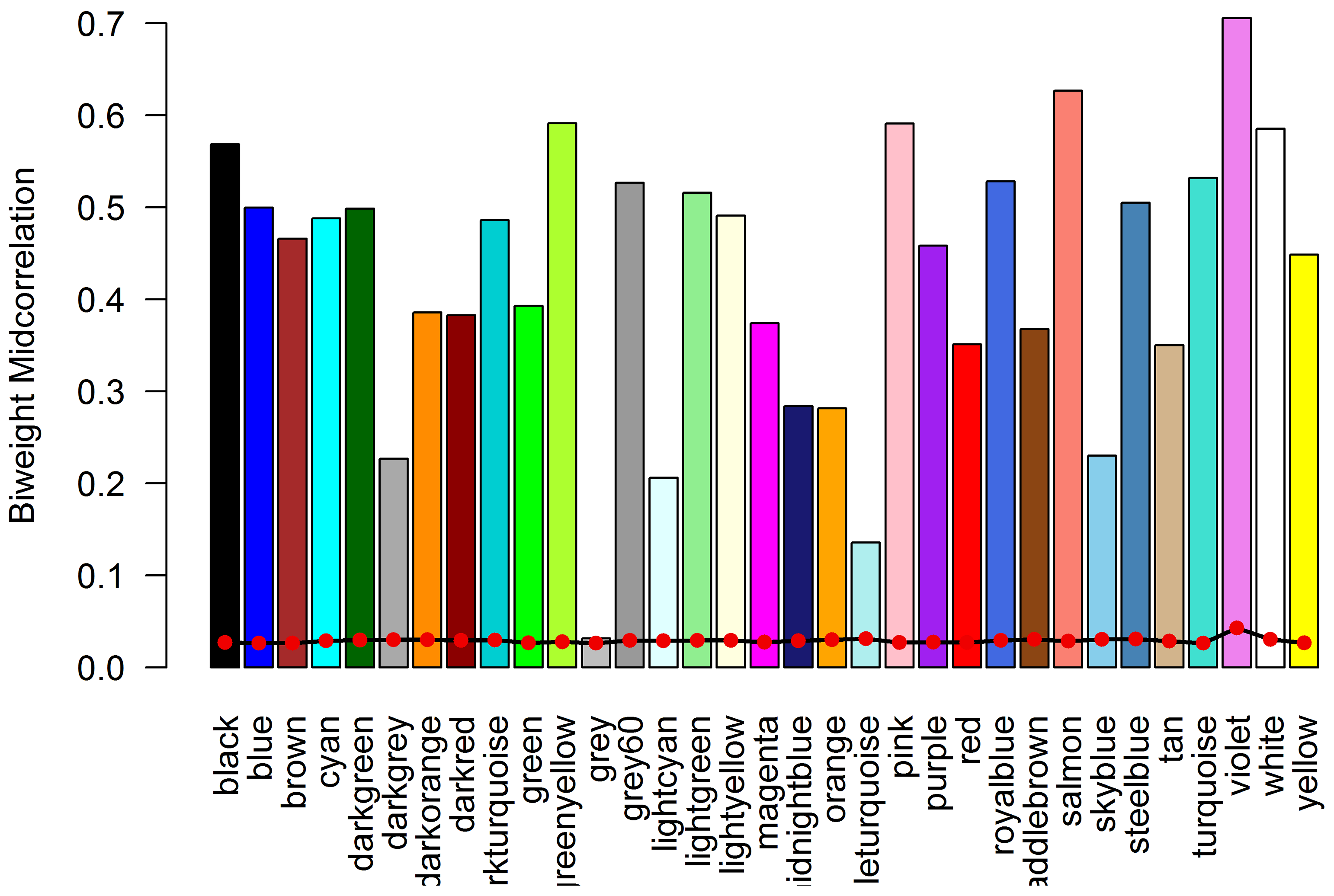

Supplement: S2 Fig — The bar plot shows the average biweight midcorrelation of all gene modules in the brain developmental network. The red circle within each bar represents the average biweight midcorrelation of 10,000 randomly selected gene sets of equal size to the genes within the respective module. (TIF) [file pone.0178532.s002.tif]

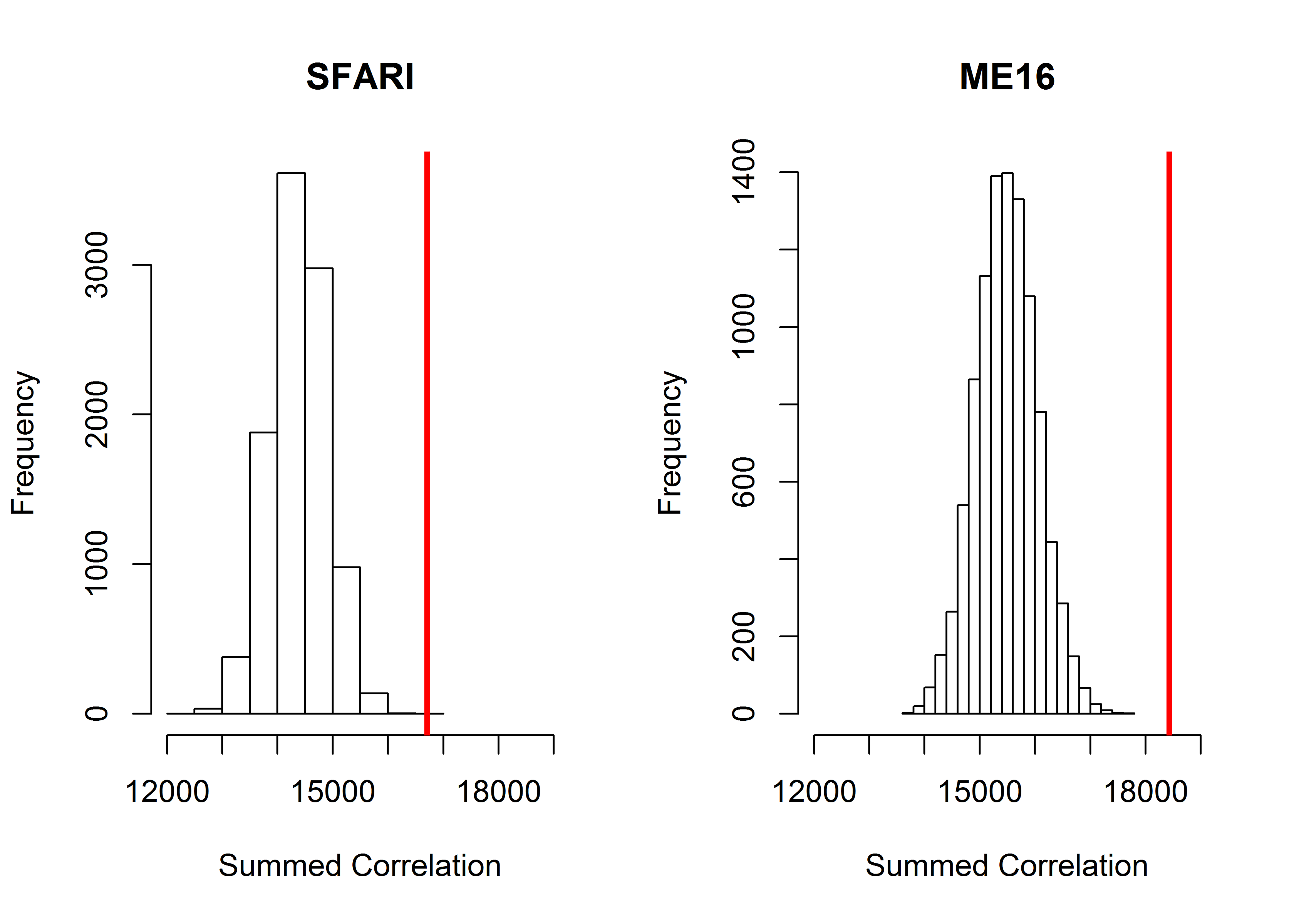

Supplement: S3 Fig — The histograms display the summed biweight midcorrelation between ASD gene sets and 10,000 randomly selected gene sets of equal size to the differentially expressed lncRNAs. The red vertical line represents the sum of the biweight midcorrelation between an ASD gene set with the differentially expressed lncRNAs. P-values were calculated based on the difference between the actual summed correlation (red line) and the permuted normal distribution and adjusted for multiple comparisons (adjusted p-values < 0.001). (TIF) [file pone.0178532.s003.tif]
