# Supplementary material for: Integrative genomic analyses for identification and prioritization of long non-coding RNAs associated with autism
Source: PLoS One. 2017 May 31;12(5):e0178532. doi: 10.1371/journal.pone.0178532 (PMC5451068; doi:10.1371/journal.pone.0178532)
Supplement: S1 Text — The information includes Gene Ontology functional enrichment of up- and down-regulated genes in the ASD cortex, and enrichment of ASD risk genes in the significantly differentially expressed genes of the ASD cortex. (DOCX) [file pone.0178532.s004.docx]

**S1 Text. Characteristics of genes differentially expressed in the ASD cortex**

1. **Gene set enrichment analysis**

Gene Ontology functional enrichment analysis was performed for the significantly up- and down-regulated genes in the ASD cortex (FDR adjusted p-value < 0.05; |Log_2_ fold change| ≥ 1) using the GOstats (R Package, v2.36), and only terms classified as biological processes are displayed [43].

1. **Up-regulated genes**

| **GOBPID** | **Pvalue** | **OddsRatio** | **ExpCount** | **Count** | **Size** | **Term** |
| --- | --- | --- | --- | --- | --- | --- |
| GO:0002376 | 1.27E-24 | 2.91 | 73.37 | 163 | 2348 | immune system process |
| GO:0007166 | 2.46E-24 | 2.81 | 81.09 | 173 | 2595 | cell surface receptor signaling pathway |
| GO:0034097 | 3.99E-20 | 3.86 | 24.43 | 78 | 782 | response to cytokine |
| GO:0006955 | 5.95E-20 | 2.99 | 47.15 | 115 | 1509 | immune response |

1. **Down-regulated genes**

| **GOBPID** | **Pvalue** | **OddsRatio** | **ExpCount** | **Count** | **Size** | **Term** |
| --- | --- | --- | --- | --- | --- | --- |
| GO:0007268 | 2.07E-28 | 5.089542 | 20.94962 | 84 | 588 | chemical synaptic transmission |
| GO:0098916 | 2.07E-28 | 5.089542 | 20.94962 | 84 | 588 | anterograde trans-synaptic signaling |
| GO:0099536 | 2.07E-28 | 5.089542 | 20.94962 | 84 | 588 | synaptic signaling |
| GO:0099537 | 2.07E-28 | 5.089542 | 20.94962 | 84 | 588 | trans-synaptic signaling |

1. **Enrichment of ASD risk genes in the differentially expressed genes**

One-sided Fisher’s exact test was performed for the overrepresentation of ASD risk genes in the significantly differentially expressed genes (p-value < 0.05; odds ratio > 1). The set of genes in the developmental co-expression network was used as the total background set (n = 26,188).

DE Not DE
 ASD 47 334
 non-ASD 1221 24586

Fisher's Exact Test for Count Data

 p-value = 4.377e-09
 alternative hypothesis: true odds ratio is not equal to 1
 95 percent confidence interval:
 2.030599 3.877353
 sample estimates:
 odds ratio
 2.833315
