# Supplementary material for: Integrative genomic analyses for identification and prioritization of long non-coding RNAs associated with autism
Source: PLoS One. 2017 May 31;12(5):e0178532. doi: 10.1371/journal.pone.0178532 (PMC5451068; doi:10.1371/journal.pone.0178532)

Integrative genomic analyses for identification and prioritization of long non-coding RNAs associated with autism

Brian L. Gudenas, Anand K. Srivastava, Liangjiang Wang

## Abstract

### Genetic studies have identified many risk loci for autism spectrum disorder (ASD) although causal factors in the majority of cases are still unknown. Currently, known ASD risk genes are all protein-coding genes; however, the vast majority of transcripts in humans are non-coding RNAs (ncRNAs) which do not encode proteins. Recently, long non-coding RNAs (lncRNAs) were shown to be highly expressed in the human brain and crucial for normal brain development. We have constructed a computational pipeline for the integration of various genomic datasets to identify lncRNAs associated with ASD. This pipeline utilizes differential gene expression patterns in affected tissues in conjunction with gene co-expression networks in tissue-matched non-affected samples. We analyzed RNA-seq data from the cortical brain tissues from ASD cases and controls to identify lncRNAs differentially expressed in ASD. We derived a gene co-expression network from an independent human brain developmental transcriptome and detected a convergence of the differentially expressed lncRNAs and known ASD risk genes into specific co-expression modules. Co-expression network analysis facilitates the discovery of associations between previously uncharacterized lncRNAs with known ASD risk genes, affected molecular pathways and at-risk developmental time points. In addition, we show that some of these lncRNAs have a high degree of overlap with major CNVs detected in ASD genetic studies. By utilizing this integrative approach comprised of differential expression analysis in affected tissues and connectivity metrics from a developmental co-expression network, we have prioritized a set of candidate ASD-associated lncRNAs. The identification of lncRNAs as novel ASD susceptibility genes could help explain the genetic pathogenesis of ASD.

### Main Objectives

1. Identify lncRNAs differentially expressed (DE lncRNAs) in the ASD cortex (SRP007483)
2. Build a brain developmental gene co-expression network (Brainspan Developmental Transcriptome)
3. Map DE lncRNAs and known ASD risk genes onto the network
4. Characterize the function and developmental expression profile of lncRNA enriched modules
5. Create a function to process ASD-associated CNVs and identify overlaps with lncRNAs
6. Filter and prioritize DE lncRNAs

### Input Data

The Input file "All_Sup_data.RData" contains:

- ASD cortex RNA-seq data: (GEO accession GSE30573)
  - **txi.salmon**: Results from using tximport on transcript quantifications (quant.sf files) produced from Salmon
  - **DEG_sample**: Sample metadata
- BrainSpan Developmental Transcriptome dataset: downloaded from "<http://www.brainspan.org/static/download.html>"
  - **Bspan_rows**: Gene level metadata (rows of Expr)
  - **clinical**: Sample metadata (cols of Expr)
  - **Expr**: Expression matrix
- ASD data : from "<https://gene.sfari.org/autdb/HG_Home.do>"
  - **SFARI**: ASD risk gene list
  - **SFARI_scores**: curated Risk scores for ASD risk gene list
  - **CNVs**: ASD CNV summary data from SFARI
  - **ME16**: ASD-associated Module gene list from Parikshak, 2013 (Table S1A)
- Gene Tissue Expression project data: from <http://www.gtexportal.org/>
  - **GTEx**: Gene-level Median RPKM across tissue types
  - **GTEx_samples**: Sample metadata

### Acknowledgments

Several elements of the code presented here are derived from the excellent resources below

<https://labs.genetics.ucla.edu/horvath/CoexpressionNetwork/Rpackages/WGCNA/Tutorials/>

<https://labs.genetics.ucla.edu/horvath/htdocs/CoexpressionNetwork/developingcortex/>

### Source Code

## R version 3.3.2

#load libraries
library(GenomicFeatures)
library(tximport)
library(readr)
library(DESeq2)
library(biomaRt)
library(WGCNA)

## ==========================================================================
## *
## * Package WGCNA 1.51 loaded.
## *
## * Important note: It appears that your system supports multi-threading,
## * but it is not enabled within WGCNA in R.
## * To allow multi-threading within WGCNA with all available cores, use
## *
## * allowWGCNAThreads()
## *
## * within R. Use disableWGCNAThreads() to disable threading if necessary.
## * Alternatively, set the following environment variable on your system:
## *
## * ALLOW_WGCNA_THREADS=<number_of_processors>
## *
## * for example
## *
## * ALLOW_WGCNA_THREADS=4
## *
## * To set the environment variable in linux bash shell, type
## *
## * export ALLOW_WGCNA_THREADS=4
## *
## * before running R. Other operating systems or shells will
## * have a similar command to achieve the same aim.
## *
## ==========================================================================

library(dplyr)
library(stringr)
library(GenomicRanges)
library(genefilter)
library(gplots)
library(RColorBrewer)
library(GOstats)
library(org.Hs.eg.db)
options(stringsAsFactors=FALSE)
enableWGCNAThreads()

## Allowing parallel execution with up to 3 working processes.

# Load in all Supplementary Input Data
load(file="./Data/All_Sup_Data.RData")

colnames(txi.salmon$counts)= rownames(DEG_samples)

# Differential Gene Expression Analysis
dds = DESeqDataSetFromTximport(txi.salmon, DEG_samples, ~Class)
dds_DE =DESeq(dds)
res05 =results(dds_DE, alpha = 0.05, contrast = c("Class","ASD","Con"))

# pdf("./Figures/MA_plot.pdf")
plotMA(res05, main="MA plot of ASD/Con fold change", ylim=c(-4,4))


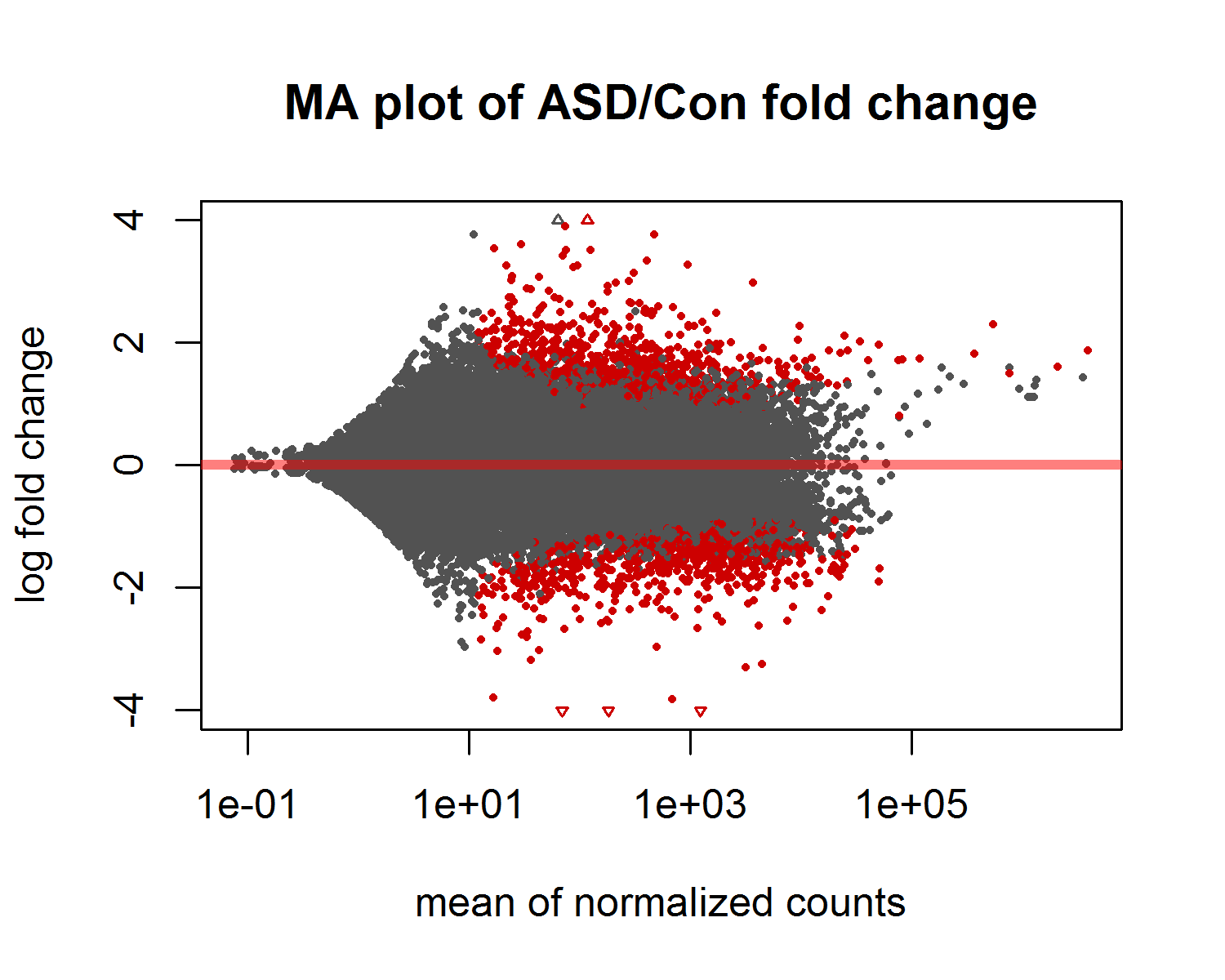


# dev.off()
res05= res05[!is.na(res05$padj), ]
rownames(res05) = unlist(lapply(strsplit(rownames(res05), "\\."),"[[",1)) ## remove trailing decimals

#Use biomaRt to exract gene biotypes
mart = useMart("ensembl", dataset = "hsapiens_gene_ensembl")
map = getBM(mart = mart, attributes=c("ensembl_gene_id","gene_biotype","start_position","end_position","chromosome_name", "external_gene_name","entrezgene"),filters = "ensembl_gene_id", values=rownames(res05))

sig_match = match(rownames(res05), map$ensembl_gene_id)
res05$gene_name = map$external_gene_name[sig_match]
res05$biotype = map$gene_biotype[sig_match]
res05$chromosome = map$chromosome_name[sig_match]
res05$start_pos = map$start_position[sig_match]
res05$end_pos = map$end_position[sig_match]
res05$entrez = map$entrezgene[sig_match]

## create filter of all lncRNA biotypes as defined by Ensembl
lncRNA_filter = c("3prime_overlapping_ncrna", "antisense","antisense RNA", "lincRNA","ncrna host","processed_transcript", "sense_intronic" , "sense_overlapping")

lnc_test=c()
for (biotype in res05$biotype) {
 lnc_test =c(lnc_test, (sum(grepl(biotype, lncRNA_filter)) > 0 ) )
}
res05$lncRNA = lnc_test

tab = data.frame(logFC = res05$log2FoldChange, negLogPval = -log10(res05$padj))

par(mar = c(5, 4, 4, 4))
plot(tab, cex = 0.6, xlab=expression(Log[2]~fold~change), pch=1,
 ylab = expression(-Log[10]~pvalue), ylim = c(0,13))
title(main = "Differentially Expressed Genes in the ASD brain")
lfc = 1
pval = 0.05
signGenes = (abs(tab$logFC) >= lfc & tab$negLogPval > -log10(pval) & !res05$lncRNA)
points(tab[signGenes, ], pch = 1, cex = 0.8, col = "red3")
signLncRNAs = (abs(tab$logFC) >= lfc & tab$negLogPval > -log10(pval) & res05$lncRNA)
points(tab[signLncRNAs, ], pch = 1, cex = 0.8, col = "blue")
abline(h = -log10(pval), col = "magenta", lty = 2, lwd = 2)
abline(v = c(-lfc, lfc), col = "green2", lty = 2, lwd= 2)
mtext(paste("pval =", pval), side = 4, at = -log10(pval), cex = 0.8, line = 0.5, las = 1)
mtext(c(paste("-", lfc, "fold"), paste("+", lfc, "fold")), side = 3, at = c(-lfc, lfc),
 cex = 0.8, line = 0.5)
legend(3, 13, c("LncRNA","Non-lncRNA"), pch=c(1,1), col=c("blue","red3"))


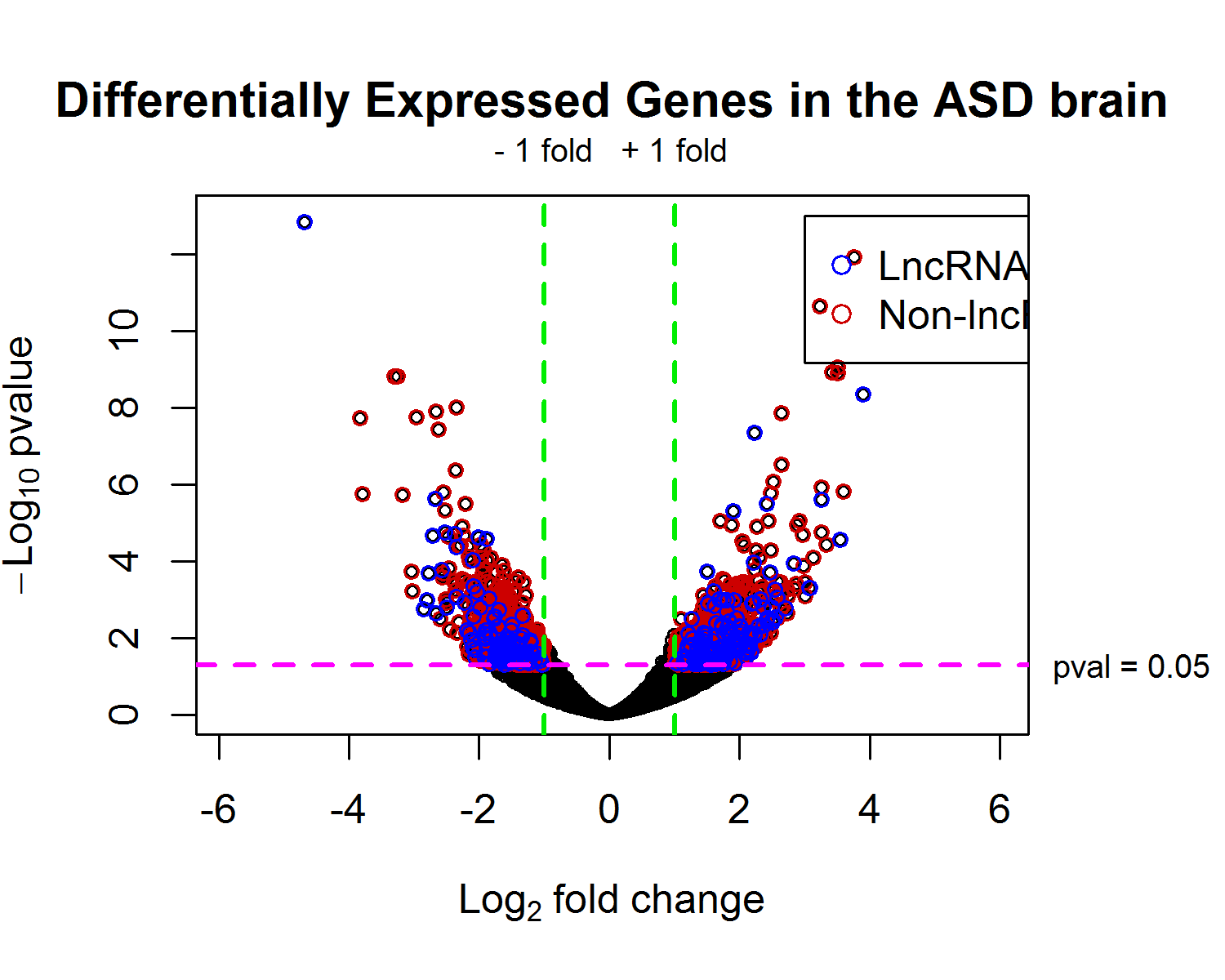


DEG =res05[res05$padj <= 0.05, ]
# Next we perform GO enrichment for the Up- and Down-regulated differentially expressed genes
paramBP_UP <- new("GOHyperGParams", geneIds = DEG$entrez[DEG$log2FoldChange > 0 ], universeGeneIds = res05$entrez_id,
 ontology = c("BP"), annotation = "org.Hs.eg", pvalueCutoff = 0.01, testDirection = "over")

## Warning in makeValidParams(.Object): removing duplicate IDs in geneIds

paramBP_Down <- new("GOHyperGParams", geneIds = DEG$entrez[DEG$log2FoldChange < 0 ], universeGeneIds = res05$entrez_id,
 ontology = c("BP"), annotation = "org.Hs.eg", pvalueCutoff = 0.01, testDirection = "over")

## Warning in makeValidParams(.Object): removing duplicate IDs in geneIds

hypUP <- hyperGTest(paramBP_UP)
hypDown <- hyperGTest(paramBP_Down)
termsUP = summary(hypUP)[1:4, ]
termsDown = summary(hypDown)[1:4, ]
termsUP #Up-regulated signficantly enriched biological processes

## GOBPID Pvalue OddsRatio ExpCount Count Size
## 1 GO:0002376 1.267844e-24 2.910216 73.37500 163 2348
## 2 GO:0007166 2.456590e-24 2.817939 81.09375 173 2595
## 3 GO:0034097 3.988314e-20 3.863335 24.43750 78 782
## 4 GO:0006955 5.946789e-20 2.998521 47.15625 115 1509
## Term
## 1 immune system process
## 2 cell surface receptor signaling pathway
## 3 response to cytokine
## 4 immune response

termsDown #Down-regulated signficantly enriched biological processes

## GOBPID Pvalue OddsRatio ExpCount Count Size
## 1 GO:0007268 2.072828e-28 5.089542 20.94962 84 588
## 2 GO:0098916 2.072828e-28 5.089542 20.94962 84 588
## 3 GO:0099536 2.072828e-28 5.089542 20.94962 84 588
## 4 GO:0099537 2.072828e-28 5.089542 20.94962 84 588
## Term
## 1 chemical synaptic transmission
## 2 anterograde trans-synaptic signaling
## 3 synaptic signaling
## 4 trans-synaptic signaling

# check # of significant DE lncRNAs
table(DEG$lncRNA)

##
## FALSE TRUE
## 1339 263

## check % of lncRNAs from each lncRNA biotype
table(DEG$biotype[DEG$lncRNA])/sum(DEG$lncRNA)*100

##
## antisense lincRNA processed_transcript
## 41.064639 45.247148 6.083650
## sense_intronic sense_overlapping
## 3.422053 4.182510

write.csv(DEG,"./Data/DEG_Results_alpha05.csv")

We have identified 263 differentially expressed lncRNAs (DE lncRNAs). Next, we will construct a weighted gene co-expression network using the BrainSpan Developmental transcriptome to infer the biological functions of these lncRNAs.

score_match = match(SFARI_scores$Gene.Symbol, SFARI$Gene.Symbol)
SFARI_scores$entrez = SFARI$Entrez.GeneID[score_match]

# convert age based on diff units to a single continous variable -- Months Post-conception (conception ~10 months)
clinical$Months.Post.Conception = 0
for (i in 1:nrow(clinical)) {
 age = as.numeric(strsplit(as.character(clinical$age[i]), " ")[[1]][1])
 unit = strsplit(as.character(clinical$age[i]), " ")[[1]][2]

 if (unit == "pcw"){ age = age/4 #convert to months
 }else if (unit == "mos") { age = age+10 ## already in months but need to add 10 months for conception
 }else if (unit == "yrs") { age = (age*12)+10}## convert years to months then add 10 for conception
 clinical$Months.Post.Conception[i]=age
}

# create unique IDs for Expr and clinical samples -------------------------
colnames(Expr)<-str_c(clinical$structure_acronym, clinical$Months.Post.Conception, clinical$gender,clinical$donor_id, sep="_")
rownames(clinical) = colnames(Expr)

# filter samples to only retain regions within the neocortex by matching " cortex" and not cerebellar cortex which is in the cerebellum and not neocortex
cortical_regions = grepl(" cortex", clinical$structure_name) & clinical$structure_name != "cerebellar cortex"
# double-check brain regions included in analysis
table(clinical$structure_name[cortical_regions])

##
## amygdaloid complex
## 0
## anterior (rostral) cingulate (medial prefrontal) cortex
## 32
## caudal ganglionic eminence
## 0
## cerebellar cortex
## 0
## cerebellum
## 0
## dorsal thalamus
## 0
## dorsolateral prefrontal cortex
## 35
## hippocampus (hippocampal formation)
## 0
## inferolateral temporal cortex (area TEv, area 20)
## 34
## lateral ganglionic eminence
## 0
## medial ganglionic eminence
## 0
## mediodorsal nucleus of thalamus
## 0
## occipital neocortex
## 0
## orbital frontal cortex
## 31
## parietal neocortex
## 0
## posterior (caudal) superior temporal cortex (area 22c)
## 36
## posteroventral (inferior) parietal cortex
## 33
## primary auditory cortex (core)
## 31
## primary motor-sensory cortex (samples)
## 5
## primary motor cortex (area M1, area 4)
## 26
## primary somatosensory cortex (area S1, areas 3,1,2)
## 26
## primary visual cortex (striate cortex, area V1/17)
## 33
## striatum
## 0
## temporal neocortex
## 0
## upper (rostral) rhombic lip
## 0
## ventrolateral prefrontal cortex
## 35

#Remove the primary motor-sensory cortex bc has only 5 samples while the rest all have ~30
cortical_regions = grepl(" cortex", clinical$structure_name) & clinical$structure_name != "cerebellar cortex" & clinical$structure_name != "primary motor-sensory cortex (samples)"

## total number of samples included for downstream analysis
sum(cortical_regions)

## [1] 352

# filter clinical and Expr by cortical_region -----------------------------
clinical = clinical[cortical_regions, ]
Expr = Expr[ ,cortical_regions]
rownames(Expr) = Bspan_rows$ensembl_gene_id

Gene_filter = rowVars(Expr) > median(rowVars(Expr))
datExpr0 = Expr[Gene_filter, ] ## variance filter to remove lowly variable genes
datExpr0 = t(datExpr0)
dim(datExpr0)

## [1] 352 26188

##--Create Genelist dataframe containing all gene info from (DEG and SFARI)
genelist = Bspan_rows[Gene_filter,]
DEG_map = match(genelist$ensembl_gene_id, rownames(DEG))

genelist$L2FC = DEG$log2FoldChange[DEG_map]
genelist$L2FC[is.na(genelist$L2FC)] = 0 ### Give 0 to any gene not Differentially expressed
## number of DEG genes not present in the filtered BrainSpan Dataset
nrow(DEG) - table(genelist$L2FC!=0)[[2]]

## [1] 252

mart = useMart("ensembl", dataset = "hsapiens_gene_ensembl")
map = getBM(mart = mart, attributes = c("ensembl_gene_id","band","gene_biotype","chromosome_name","start_position","end_position"),
 filters = "ensembl_gene_id", values = genelist$ensembl_gene_id)

mart_map = match(genelist$ensembl_gene_id, map$ensembl_gene_id)

genelist$biotype = map$gene_biotype[mart_map]
genelist$chromosome = map$chromosome_name[mart_map]
genelist$start = map$start_position[mart_map]
genelist$end = map$end_position[mart_map]

ASD_match = match(genelist$entrez_id, SFARI_scores$entrez)
genelist$ASD_score = SFARI_scores$Score[ASD_match]

lnc_test=c()
for (biotype in genelist$biotype) {
 lnc_test =c(lnc_test, (sum(grepl(biotype, lncRNA_filter)) > 0))
}
genelist$lncRNA = lnc_test

LncRNA_Gr = makeGRangesFromDataFrame(genelist[genelist$lncRNA == TRUE & genelist$L2FC != 0, colnames(genelist)=="chromosome" | colnames(genelist)=="start" | colnames(genelist)=="end" ])

genelist_Gr = makeGRangesFromDataFrame(genelist[genelist$lncRNA != TRUE & !is.na(genelist$start), colnames(genelist)=="chromosome" | colnames(genelist)=="start" | colnames(genelist)=="end" ], start.field = "start", end.field = "end" )
names(genelist_Gr) = genelist$gene_symbol[genelist$lncRNA != TRUE & !is.na(genelist$start) ]

near_genes = names(genelist_Gr)[nearest(x = LncRNA_Gr, subject = genelist_Gr)]
genelist$DEG_lncRNA_nearest_gene[genelist$lncRNA == TRUE & genelist$L2FC != 0] = near_genes
genelist$nearest_ASD_score[genelist$lncRNA == TRUE & genelist$L2FC != 0] = as.character(genelist$ASD_score[match( near_genes, genelist$gene_symbol)])
## Check how many DE lncRNAs nearest neighbor genes are ASD risk genes
genelist[genelist$lncRNA==TRUE & genelist$L2FC !=0 & !is.na(genelist$nearest_ASD_score), -c(1,2,3,5,11,12)]

## gene_symbol L2FC biotype chromosome start end
## 28724 AC127496.3 -1.199485 lincRNA 17 81029130 81034881
## 30134 AC018712.3 -1.892773 antisense 2 172677141 172736206
## 32955 DLX6-AS1 -1.366135 antisense 7 96955141 97014065
## 34241 RP11-497D6.4 -1.882004 antisense 6 146841901 147204614
## 48645 RP11-867G23.2 -4.675621 antisense 11 66267635 66268129
## 52229 RP11-707P17.2 1.873206 antisense 15 51457286 51460582
## DEG_lncRNA_nearest_gene nearest_ASD_score
## 28724 BAIAP2 5
## 30134 RAPGEF4 4
## 32955 DLX6 4
## 34241 STXBP5 3
## 48645 KLC2 5
## 52229 DMXL2 4

table(rownames(clinical)==rownames(datExpr0)) ## verify clinical matches expression for all samples

##
## TRUE
## 352

gsg = goodSamplesGenes(datExpr0,verbose=4);

## Flagging genes and samples with too many missing values...
## ..step 1

if (!gsg$allOK)
{
 if (sum(!gsg$goodGenes)>0)
 printFlush(paste("Removing genes:", paste(colnames(datExpr0)[!gsg$goodGenes], collapse = ", ")));
 if (sum(!gsg$goodSamples)>0)
 printFlush(paste("Removing samples:", paste(rownames(datExpr0)[!gsg$goodSamples], collapse = ", ")))
 datExpr0= datExpr0[gsg$goodSamples, gsg$goodGenes]
 genelist=genelist[gsg$goodGenes,]
}

datExpr0 <- log2(datExpr0+1)

powers = c(seq(8,14,by=1), seq(14,26, by=2));
rm(Expr, tab, txi.salmon, res05, dds, dds_DE, gsg, mart_map, near_genes, signLncRNAs, signGenes, Gene_filter, genelist_Gr)

save.image("./Data/pre_network.RData")

Now we will create the gene co-expression network. It is important that this next code block is run on a machine with at least 16GB of RAM.

load("./Data/pre_network.RData")

sft=pickSoftThreshold(datExpr0, powerVector=powers, verbose=5, networkType="signed", corFnc = "bicor",corOptions = list(use = 'p', maxPOutliers = 0.1), blockSize = 30000)

sft$fitIndices
# Power SFT.R.sq slope truncated.R.sq mean.k. median.k. max.k.
# 1 8 0.7484369 -1.163762 0.9585420 774.88152 633.576486 2439.8020
# 2 9 0.7875009 -1.213461 0.9592868 598.57372 461.356967 2140.1732
# 3 10 0.8113660 -1.270069 0.9539941 472.29742 341.101313 1896.2786
# 4 11 0.8322182 -1.304654 0.9551663 379.41399 256.678283 1693.9240
# 5 12 0.8469340 -1.336352 0.9581380 309.49700 195.451957 1523.4980 <- use default since SFT.R.sq >= .8
# 6 13 0.8462191 -1.375997 0.9511506 255.80322 150.457653 1378.1721
# 7 14 0.8525415 -1.398139 0.9519997 213.84419 116.805717 1252.9491
# 8 14 0.8525415 -1.398139 0.9519997 213.84419 116.805717 1252.9491
# 9 16 0.8731235 -1.425725 0.9606139 153.78549 72.083334 1048.6968
# 10 18 0.8705945 -1.459732 0.9588920 114.10436 46.139487 890.7522
# 11 20 0.8802740 -1.468286 0.9671540 86.81553 30.258156 766.7518
# 12 22 0.8859710 -1.478676 0.9697918 67.43372 20.337430 666.3259
# 13 24 0.8889207 -1.492078 0.9734972 53.29729 13.810368 583.7195
# 14 26 0.8951830 -1.497883 0.9769967 42.75418 9.531669 514.8790


net = blockwiseModules(datExpr0, power = 12,
 networkType="signed", minModuleSize = 50, maxBlockSize = 30000,
 mergeCutHeight = 0.15, deepsplit=4, corType= "bicor",corOptions = list(use = 'p', maxPOutliers = 0.1),
 numericLabels = TRUE, pamRespectsDendro = FALSE,
 saveTOMs = TRUE,
 saveTOMFileBase = "./Data/TOM",
 verbose = 3 )
save.image("./Data/Network.RData")

Next we will validate the co-expression modules to ensure they are significantly co-expressed, warning this code chunk is kind of slow.

load(file="./Data/Network.RData")
moduleColors = labels2colors(net$colors)
genelist$Module = moduleColors
mod_counts = as.numeric(table(moduleColors))
Real_coexp = data.frame(matrix(nrow=length(mod_counts), ncol = 1, data=0))
colnames(Real_coexp) = "Correlation"
rownames(Real_coexp) = names(table(genelist$Module))
for (mod in names(table(genelist$Module))) {

 actual_coexp = mean(bicor(datExpr0[ ,genelist$Module == mod], datExpr0[ ,genelist$Module == mod], use ='p', maxPOutliers = 0.1))
 Real_coexp[rownames(Real_coexp)==mod, 1] = actual_coexp
}

cor_mat=bicor(datExpr0,datExpr0 , use ='p', maxPOutliers = 0.1)
Iter = 10000
mod_counts = as.numeric(table(moduleColors))
Results = matrix(nrow=length(mod_counts), ncol = Iter, data=0)
rownames(Results) = names(table(moduleColors))

for (I in 1:Iter){
 avail_genes = 1:length(moduleColors)
 for (mod in 1:length(mod_counts)) {
 rand_genes = sample(avail_genes, mod_counts[mod])
 avail_genes = avail_genes[-rand_genes] ## remove chosen genes from available

 rand_sum = mean(cor_mat[rand_genes,rand_genes])
 #rand_sum = mean(cor(datExpr0[,rand_genes], datExpr0[,rand_genes]))
 Results[mod,I] = rand_sum
 }
}
rm(cor_mat)
Pvals =c()
for (i in 1:nrow(Results)){
 ### statistical Enrichment
 Z_scores = scale(c(as.numeric(Results[i,]),Real_coexp[i,])) ## transform all permuted and Real data to Z-scores
 Real = tail(Z_scores, 1)
 Pvals=c(Pvals, 2*pnorm(-abs(Real))) ## use pnorm to calculate P-value of real Z-score and append
 }
Pvals = p.adjust(Pvals, "fdr") ## adjust P-values for multiple testing
table(Pvals < .0001) ## check if all modules are significant

##
## TRUE
## 33

df.bar = barplot(Real_coexp$Correlation, col=rownames(Real_coexp), ylab = "Biweight Midcorrelation", main="Average Modular Coexpression", las=2, names.arg = rownames(Real_coexp))
lines(x= df.bar, y = as.numeric(rowMeans(Results)), lwd=2)
points(x= df.bar, y = as.numeric(rowMeans(Results)), col="red2", pch=16, bg="black")


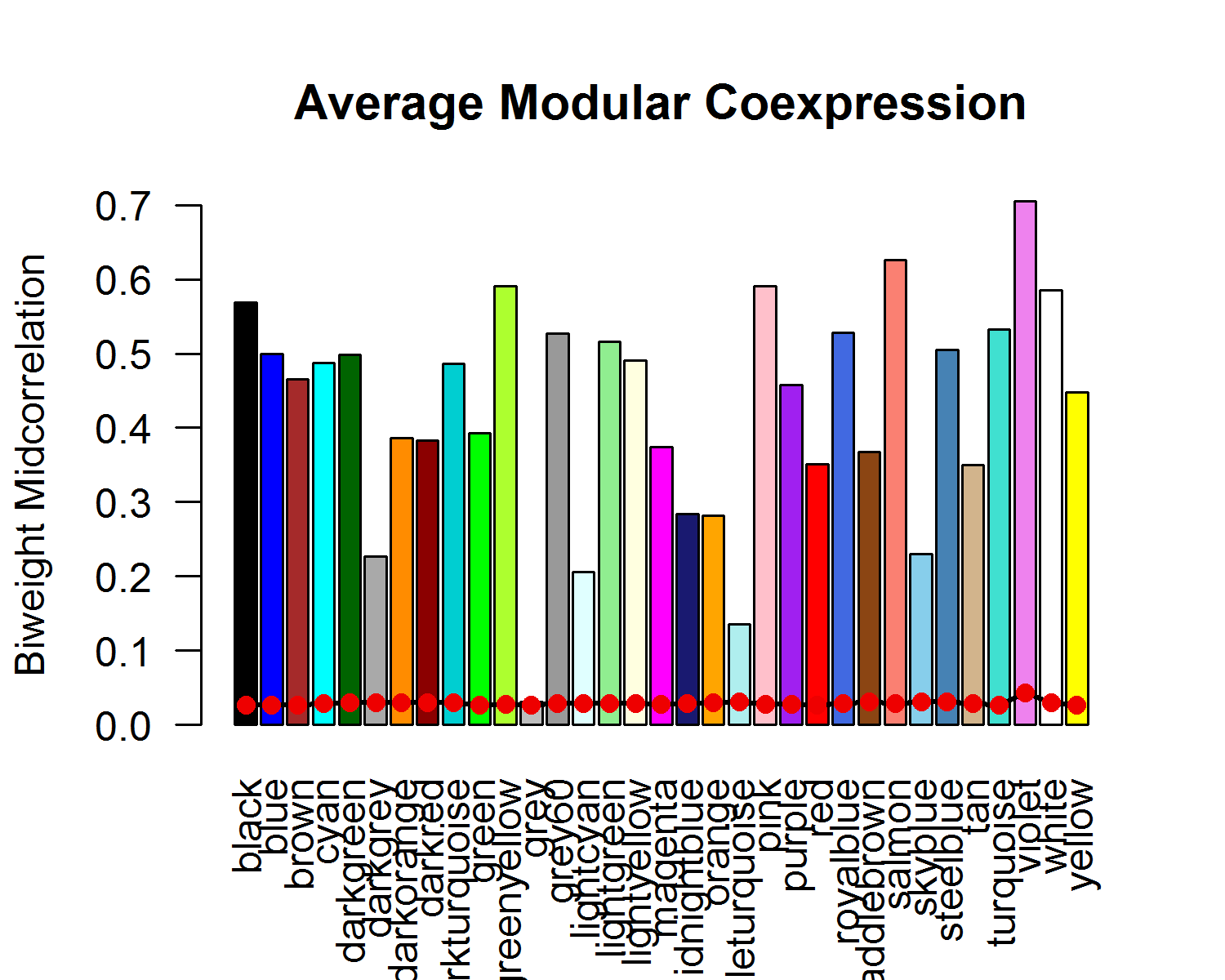


rm(Results, Real_coexp)

Now we can examine the modules and find which ones are enriched for ASD risk genes and/or DE lncRNAs.

table(net$colors)

##
## 0 1 2 3 4 5 6 7 8 9 10 11 12 13 14
## 2985 4764 2863 2851 1635 1125 957 890 733 690 593 542 384 370 350
## 15 16 17 18 19 20 21 22 23 24 25 26 27 28 29
## 330 312 307 298 294 290 280 266 264 237 236 232 224 216 214
## 30 31 32
## 208 190 58

lncrnaColors = rep("grey", nrow(genelist))
DEGcolors = rep("grey", nrow(genelist))
ASDcolors = rep("grey", nrow(genelist))
lncrnaColors[genelist$lncRNA == TRUE] = "red3"
DEGcolors[genelist$L2FC != 0 ] = "blue3"
ASDcolors[!is.na(genelist$ASD_score) ] = "black"

plotDendroAndColors(net$dendrograms[[1]], cbind(moduleColors,lncrnaColors, DEGcolors, ASDcolors),
 c("Module","LncRNAs","DEG", "ASD"),
 dendroLabels = FALSE, hang = 0.03,
 addGuide = TRUE, guideHang = 0.05)


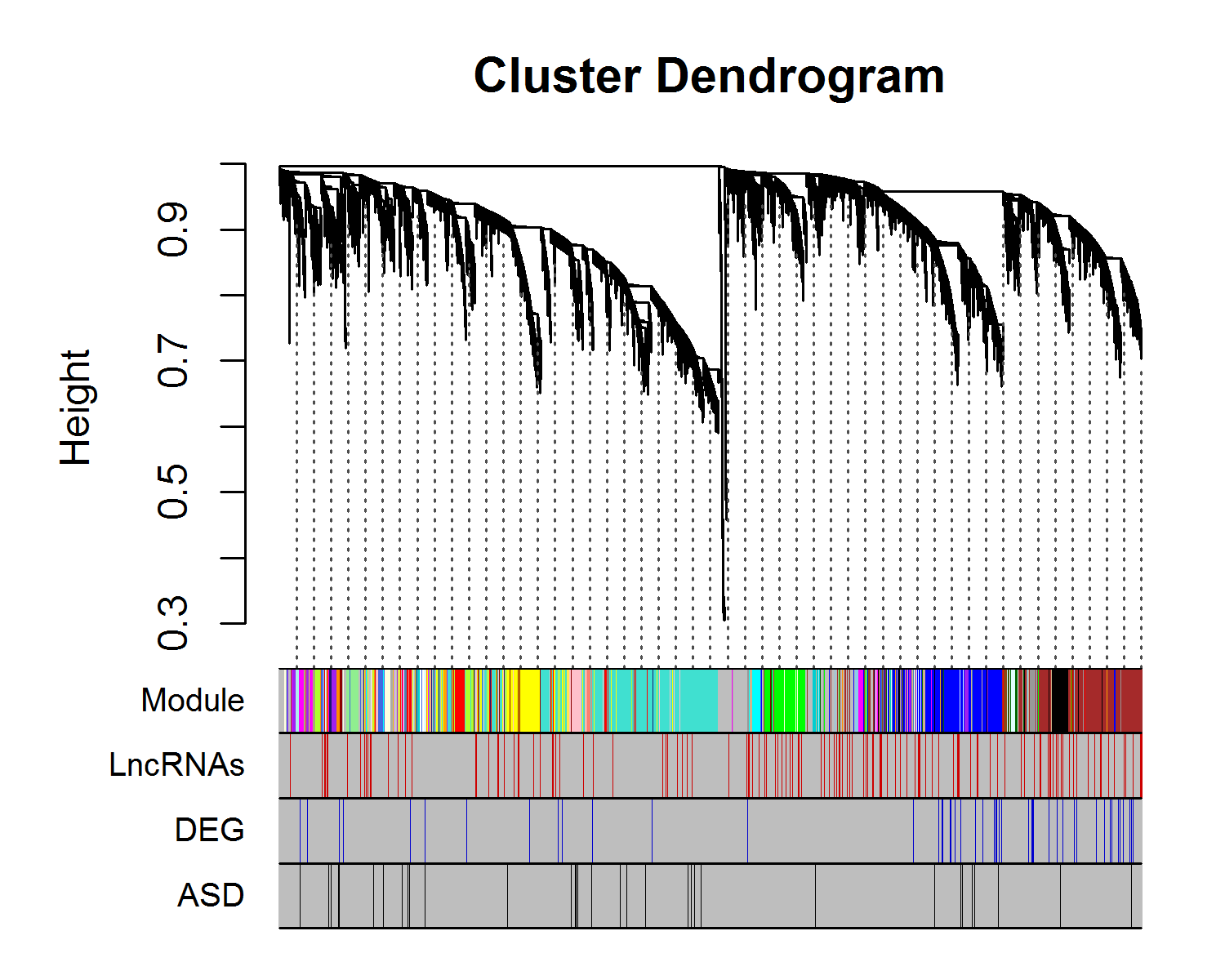


moduleLabels = net$colors

MEs = net$MEs;
geneTree = net$dendrograms[[1]];
nGenes = ncol(datExpr0)
nSamples=nrow(datExpr0)

## calculate MEs with module colors
MEs0 <-moduleEigengenes(datExpr0,moduleColors)$eigengenes
MEs = orderMEs(MEs0)

counts = model.matrix( ~structure_acronym - 1, data = clinical)
colnames(counts) = str_replace(colnames(counts), pattern = "structure_acronym", replacement = "")
counts =counts[, colSums(counts)!=0]

clinical = cbind(clinical, counts)
clinical$Male = rep(0, nrow(clinical))
clinical$Male[clinical$gender=="M"] = 1
moduleTraitCor = cor(MEs, clinical[ ,c(8:ncol(clinical))], use = "p");
moduleTraitPvalue = corPvalueStudent(moduleTraitCor, nSamples);

textMatrix = paste(signif(moduleTraitCor, 2), "\n(",
 signif(moduleTraitPvalue, 1), ")", sep = "");
dim(textMatrix) = dim(moduleTraitCor)

par(mar=c(3,8,2,1))
labeledHeatmap(Matrix = moduleTraitCor,
 xLabels = c("Months", colnames(clinical)[9:ncol(clinical)]),
 yLabels = names(MEs),
 ySymbols = names(MEs),
 colorLabels = FALSE,
 colors = greenWhiteRed(60),
 textMatrix = textMatrix,
 setStdMargins = FALSE,
 cex.text = 0.5,
 zlim = c(-1,1),
 main = paste("Module-trait relationships") )

## Warning in greenWhiteRed(60): WGCNA::greenWhiteRed: this palette is not suitable for people
## with green-red color blindness (the most common kind of color blindness).
## Consider using the function blueWhiteRed instead.

age=as.data.frame(clinical$Months.Post.Conception)
names(age)="Months_Age"
modNames=substring(names(MEs),3)
geneModuleMembership = as.data.frame(cor(datExpr0, MEs, use = "p"));

genes<-colnames(datExpr0)
genes2annot <- match(genes, genelist$ensembl_gene_id)
sum(is.na(genes2annot))

## [1] 0

MMPvalue = as.data.frame(corPvalueStudent(as.matrix(geneModuleMembership), nSamples));
names(geneModuleMembership) = paste("MM", modNames, sep="");
names(MMPvalue) = paste("p.MM", modNames, sep="");
geneTraitSignificance = as.data.frame(cor(datExpr0, age, use = "p"));
GSPvalue = as.data.frame(corPvalueStudent(as.matrix(geneTraitSignificance), nSamples));

names(geneTraitSignificance) = paste("GS.", names(age), sep="");
names(GSPvalue) = paste("p.GS.", names(age), sep="");

geneInfo= data.frame(Ensembl_ID = genelist$ensembl_gene_id,
 gene_symbol= genelist$gene_symbol,
 Log2FC_DE = genelist$L2FC,
 lncRNA=genelist$lncRNA,
 ASD=genelist$ASD_score,
 moduleColor = moduleColors,
 geneTraitSignificance,
 GSPvalue)
modOrder = order(-abs(cor(MEs, age, use = "p")));

for (mod in 1:ncol(geneModuleMembership))
{
 oldNames = names(geneInfo)
 geneInfo = data.frame(geneInfo, geneModuleMembership[, modOrder[mod]],
 MMPvalue[, modOrder[mod]]);
 names(geneInfo) = c(oldNames, paste("MM.", modNames[modOrder[mod]], sep=""),
 paste("p.MM.", modNames[modOrder[mod]], sep=""))
}
geneOrder = order(geneInfo$moduleColor, -abs(geneInfo$GS.Months_Age));
geneInfo = geneInfo[geneOrder, ]
write.csv(geneInfo, file = "./Data/geneInfo.csv",row.names = FALSE )

genelist$ASD_score = as.character(genelist$ASD_score)
genelist$ASD_score[is.na(genelist$ASD_score)]=0

moduleMembership = vector(mode = "numeric", length = nrow(geneInfo))
for (i in 1:nrow(geneInfo)){
 module = paste0("MM.",geneInfo$moduleColor[i])
 moduleMembership[i] = select(geneInfo, match(module, colnames(geneInfo)))[i,]
}
geneInfo$Membership = moduleMembership
genelist$membership =geneInfo$Membership[match(genelist$ensembl_gene_id, geneInfo$Ensembl_ID)]
save.image("./Data/Post_geneinfo_network.RData")

my_palette <- colorRampPalette(c("dark green","green","white","white","red","dark red"))(n = 599)

Module_dist = cor(MEs0,MEs0)
Modules0= paste(paste0(toupper(substr(names(table(moduleColors)), 1, 1)), tolower(substring(names(table(moduleColors)), 2))))
rownames(Module_dist) = Modules0
colnames(Module_dist) = Modules0

heatmap.2(Module_dist,trace="none",main="Module Correlation Matrix",RowSideColors=names(table(moduleColors)),notecol="black",key=TRUE,col=my_palette,symm=F,symkey=F,symbreaks=F)


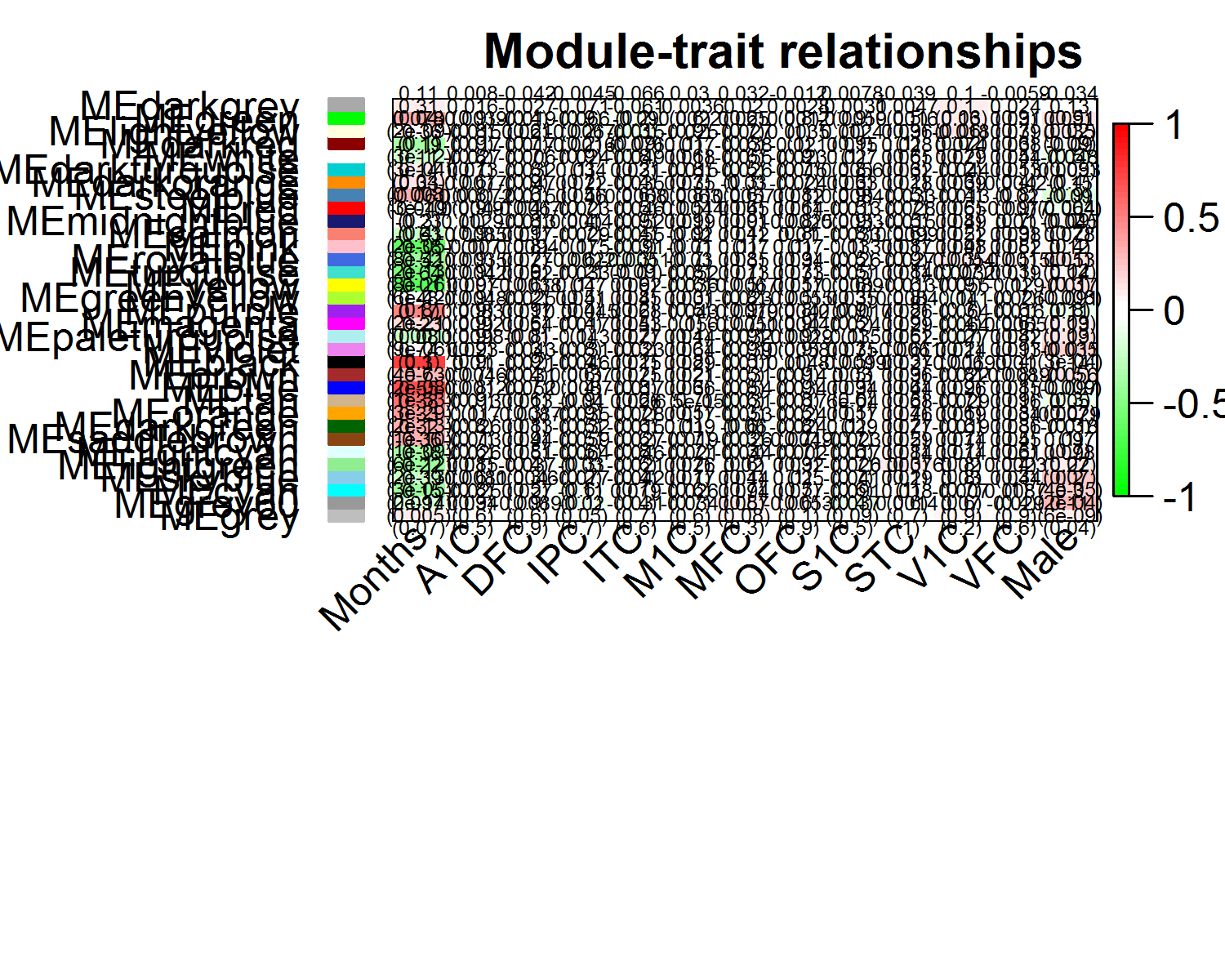

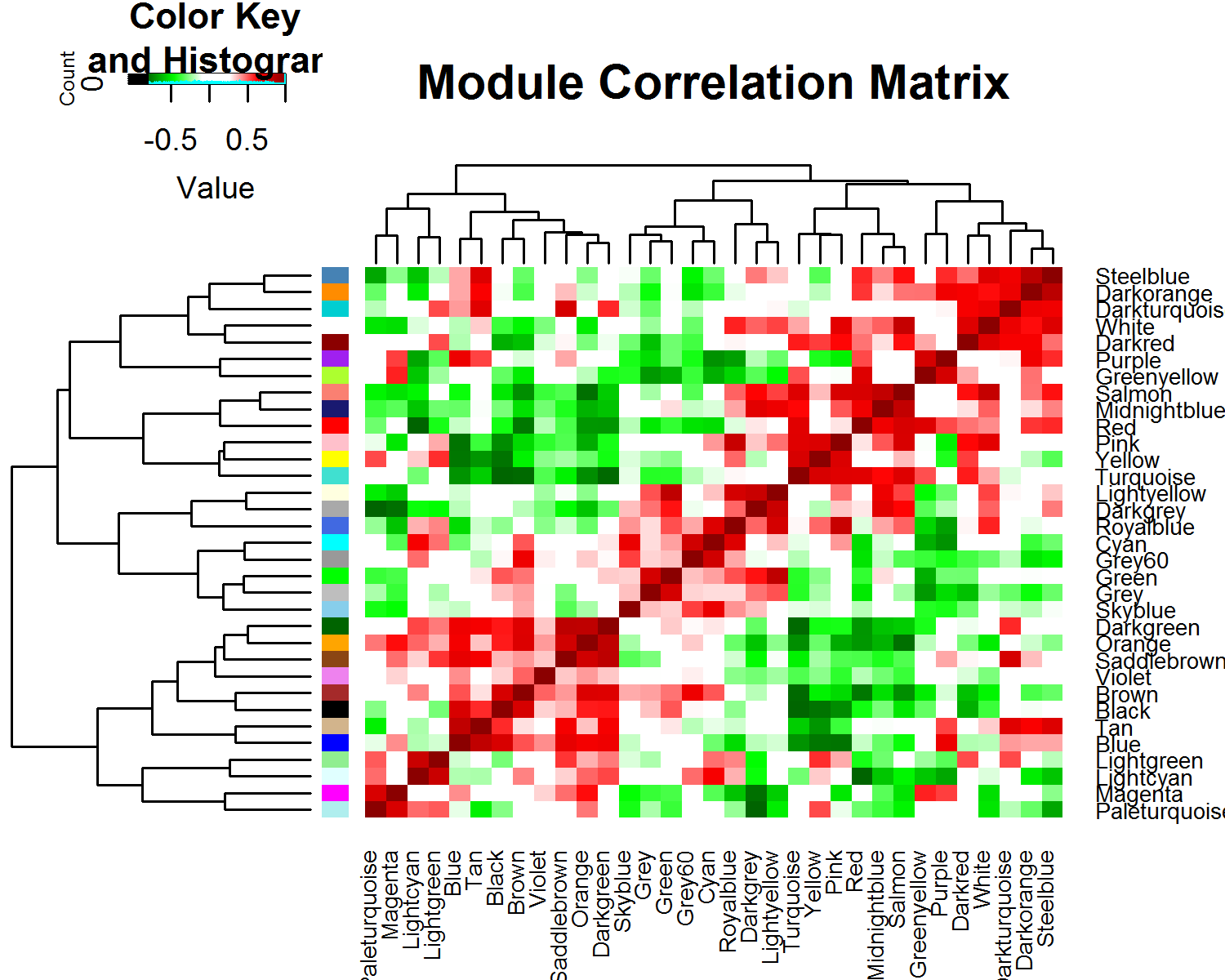


###### this code is for modular analysis of ASD genes and lncRNAS
genelist$Module = moduleColors
mod_sum<-matrix(data=NA,nrow=length(table(moduleColors)),ncol=4)
colnames(mod_sum) <- c("DE_lncRNAs","SFARI ASD","ME16","total")
rownames(mod_sum) <- names(table(moduleColors))

ME16_match = match(genelist$ensembl_gene_id, ME16)
genelist$ME16=0
genelist$ME16[!is.na(ME16_match)]=1

LncRNAs_DE = as.list(table(factor(moduleColors[genelist$lncRNA== TRUE & genelist$L2FC != 0],lev=rownames(mod_sum))))
mod_sum[,1] = as.numeric(LncRNAs_DE)

SFARI = table(factor(moduleColors[genelist$ASD_score=="1S" | genelist$ASD_score=="2S" | genelist$ASD_score=="3S" | genelist$ASD_score=="4S" | genelist$ASD_score==1 | genelist$ASD_score==2 | genelist$ASD_score==3 | genelist$ASD_score==4 | genelist$ASD_score==5],lev=rownames(mod_sum)))
mod_sum[,2] = as.numeric(SFARI)

ME16 = table(factor(moduleColors[genelist$ME16==1],lev=rownames(mod_sum)))
mod_sum[,3] = as.numeric(ME16)

mod_totals <- as.list(table(moduleColors))
mod_sum[,4]= as.numeric(mod_totals)

mod_sum = mod_sum[mod_sum[,1] > 1, ] ## remove modules with no lncRNAs

total_genes = nGenes
mat_p = matrix(data=NA, nrow=nrow(mod_sum), ncol=3)
mat_or = matrix(data=NA, nrow=nrow(mod_sum), ncol=3)
for (row in 1:nrow(mod_sum)){
 for (col in 1:3) {
 mod_total <- as.numeric(mod_sum[row, 4])
 mod_count <- as.numeric(mod_sum[row, col])
 mod_non <- mod_total-mod_count
 non_count <- sum(as.numeric(mod_sum[,col])) - mod_count
 non_non <- (total_genes-mod_total)-non_count

 contigency <- matrix(c(mod_count, mod_non, non_count, non_non),2,2)
 colnames(contigency) = c(rownames(mod_sum)[row], paste0("Non-", rownames(mod_sum)[row]))
 rownames(contigency) = c(colnames(mod_sum)[col], paste0("Non-", colnames(mod_sum)[col]))

 results <- fisher.test(contigency, alternative = "greater")
 p_val <- results[[1]]
 OR <- results[[3]]

 mat_p[row,col]<-p_val
 mat_or[row,col]<-OR
 }
}

rownames(mat_p)<-rownames(mod_sum)
rownames(mat_or)<-rownames(mod_sum)
colnames(mat_p)<-colnames(mod_sum)[1:3]
colnames(mat_or)<-colnames(mod_sum)[1:3]
### now adjust P-values for all comparisons made
 ad_p = p.adjust(mat_p, method = "fdr")
dim(ad_p) = dim(mat_p)
colnames(ad_p) <-colnames(mat_p)
rownames(ad_p) <- rownames(mat_p)
OR_filter<-matrix(data=NA,ncol=3,nrow=nrow(ad_p))
### * = p-value < 0.05 || ** = FDR-adjusted p-value < 0.05 ##### both need OR >= 1
OR_filter[ad_p <= 0.05] <- "*"
OR_filter[mat_or >= 1 & ad_p <= 0.05] <- paste0(round(mat_or[mat_or >= 1 & ad_p <= 0.05 ],2))
# log transform
ad_p<-log10(ad_p)*-1
rownames(OR_filter)<-rownames(ad_p)
my_palette <- colorRampPalette(c("white","orange","red"))(n = 399)
 Modules_cap = paste0(toupper(substr(rownames(ad_p), 1, 1)), tolower(substring(rownames(ad_p), 2)))
rownames(ad_p) = Modules_cap

heatmap.2(ad_p, cellnote=OR_filter, trace="none", main="Module Gene Set Enrichment", RowSideColors=Modules_cap, notecol="black",key=TRUE,dendrogram = "none", col=my_palette, symm=F, symkey=F, symbreaks=F, key.xlab="-Log( FDR adjusted p-value )", notecex =1.5, margins =c(13,8), breaks = seq(0, 15, length.out =400))


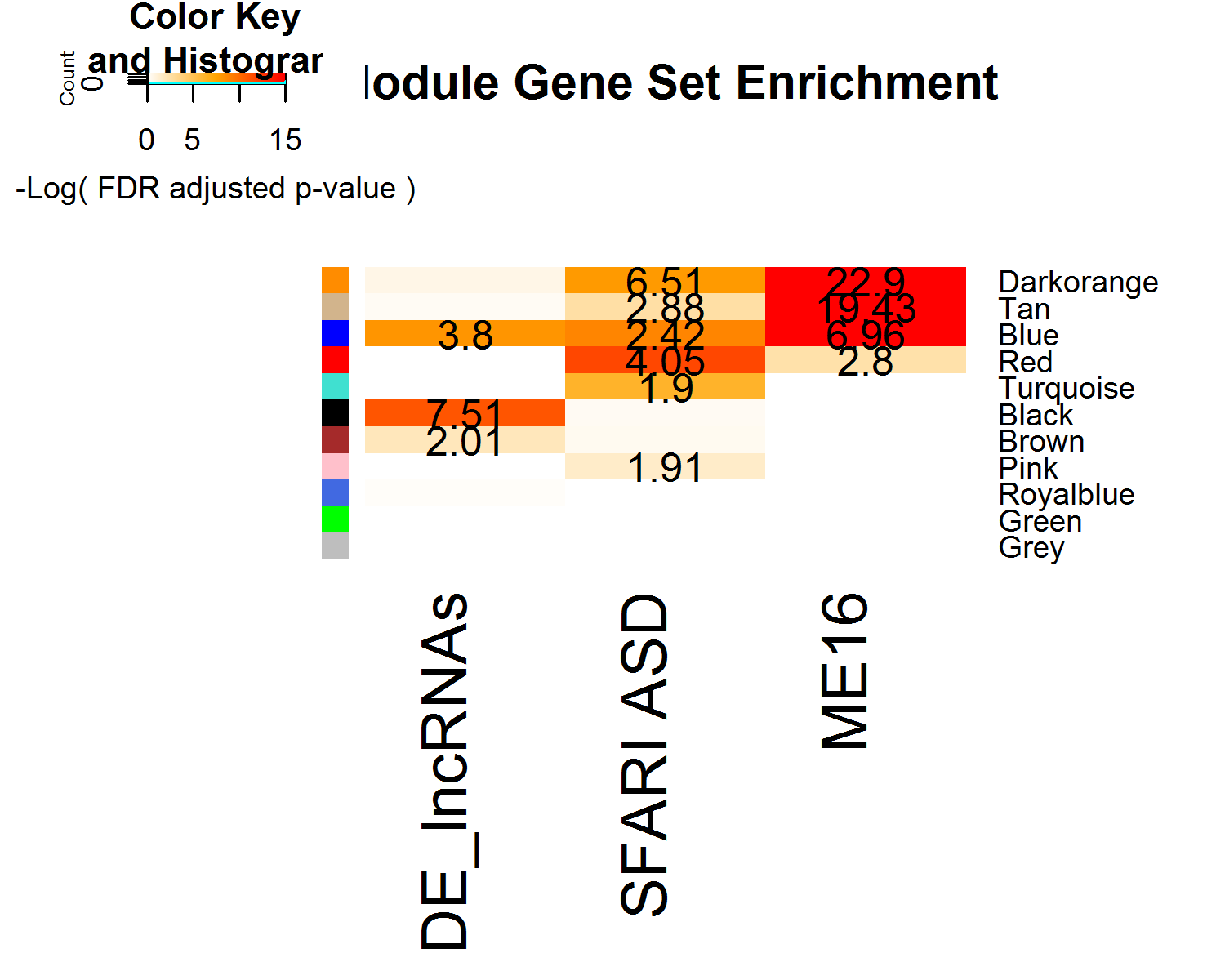


We analyze the averaged gene Log2 Fold-change (L2FC) from the ASD cortex of the module gene lists created in the developmental co-expression network. Any gene in the developmental network not considered differentially expressed in the ASD cortex has a log2 fold-change of 0, indicating the average L2FC of modules not dysregulated in the ASD cortex should approximate 0.

genesbyMod = group_by(genelist, Module)
Mod_DE = summarize(genesbyMod, Avg=mean(L2FC))
Mod_DE = Mod_DE[order(abs(Mod_DE$Avg), decreasing = TRUE), ]
### Permutation testing
Iter = 10000
 #genesbyMod = group_by(genelist, Module)
 mod_counts = as.numeric(table(moduleColors))
 Results = matrix(nrow=length(mod_counts), ncol = Iter, data=0)
 rownames(Results) = names(table(moduleColors))

 for (I in 1:Iter){
 avail_genes = 1:nrow(genelist) ##establish available genes
 for (mod in 1:length(mod_counts)) {
 rand_genes = sample(avail_genes, mod_counts[mod])
 avail_genes=avail_genes[-(rand_genes)] ## remove chosen genes from available
 rand_avg = mean(genelist$L2FC[rand_genes])
 Results[mod,I] = rand_avg
 }
 }

Pvals =c()
for (i in 1:nrow(Results)){
 ### statistical Enrichment
 Z_scores = scale(c(as.numeric(Results[i, ]), as.numeric(Mod_DE[i,2]) )) ## transform all permuted and Real data to Z-scores
 Real = tail(Z_scores, 1)
 Pvals=c(Pvals, 2*pnorm(-abs(Real))) ## use pnorm to calculate two-sided P-value of real Z-score and append
}
Pvals = p.adjust(Pvals, method = "fdr") ## adjust P-values for multiple testing

Sig_P = rep("", length(Pvals))
Sig_P[Pvals < 0.05]="*"
## reorder Sig_P, Mod_DE and Results by Mod_DE
Results = Results[order(abs(Mod_DE$Avg), decreasing = TRUE),]
Sig_P = Sig_P[order(abs(Mod_DE$Avg), decreasing = TRUE)]
Mod_DE = Mod_DE[order(abs(Mod_DE$Avg), decreasing = TRUE),]
Sig_pos = rep(1, length(Sig_P)) ##create position vector for sig asterisks
Sig_pos[Mod_DE$Avg > 0] =3
par(mar = c(6.5, 4, 3, 3));
df.bar = barplot(Mod_DE$Avg, col=Mod_DE$Module, ylab = "Log2 Fold Change", main="Differential Expression of Modules in ASD", las=2, names.arg = Mod_DE$Module, ylim=range(-0.25,0.25))
lines(x= df.bar, y = as.numeric(rowMeans(Results)))
points(x= df.bar, y = as.numeric(rowMeans(Results)), col="red", pch=16)
text(x= df.bar,Mod_DE$Avg-.008, Sig_P, cex =2, pos=Sig_pos, col="black", offset = 0)


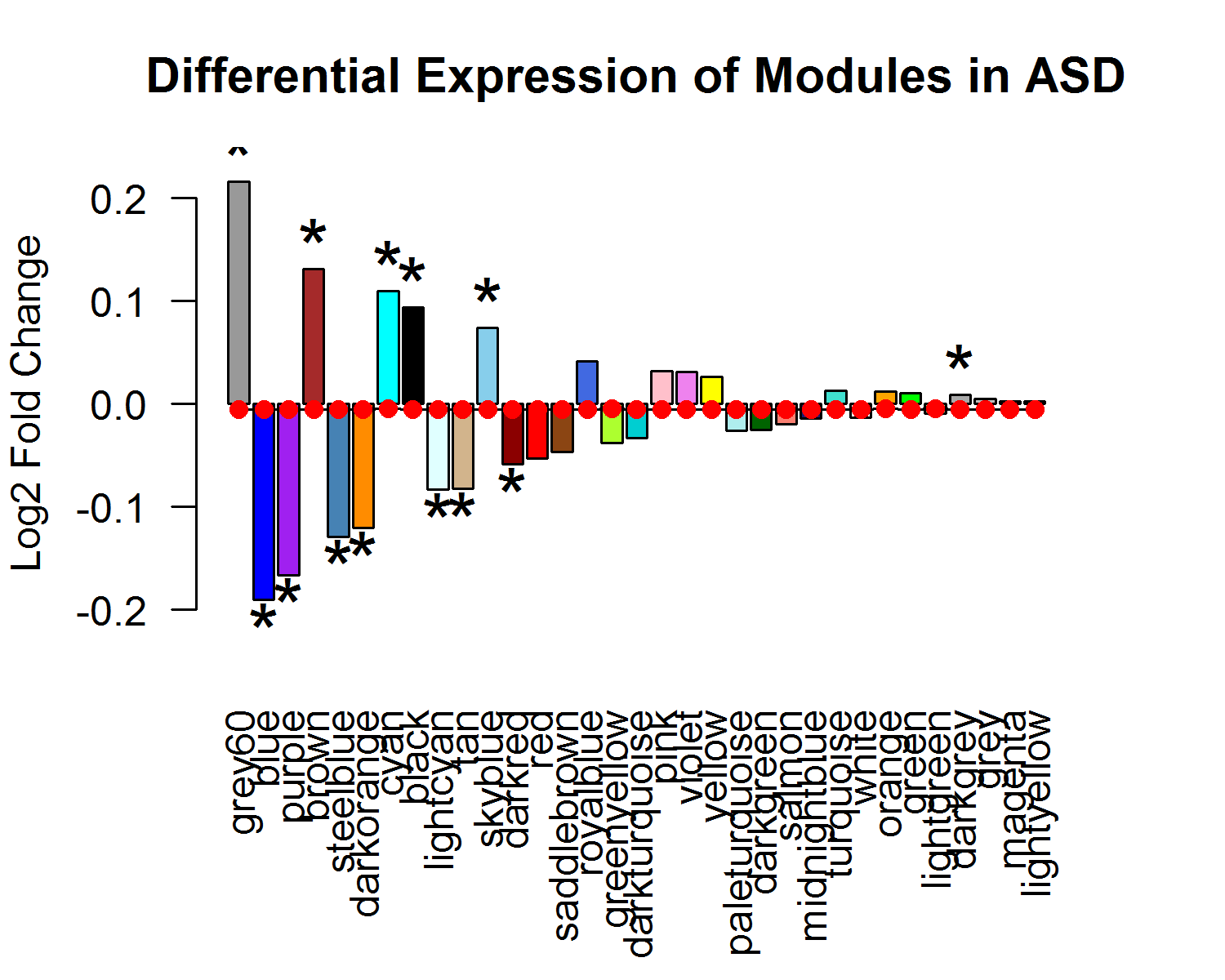


## white asterisks denote significance at p = 0.05

Next we perform co-expression permutations to quantify the strength to which DE lncRNAs are co-expressed with ASD risk genes.

lncRNAlist = genelist[genelist$lncRNA & genelist$L2FC !=0, ]

ASD_lncMatch = match( lncRNAlist$ensembl_gene_id , colnames(datExpr0))
genelist$ASD_score[is.na(genelist$ASD_score)] = 0
ASD_genes = genelist$ASD_score != 0 & genelist$ASD_score != "S" & genelist$ASD_score != "6" & genelist$ASD_score != "5"
ME16_genes = genelist$ME16==1
gene_logicals= list(ASD_genes,ME16_genes)
titles =c("SFARI", "ME16")

par(mfrow=c(1,2))
j=1
for ( i in gene_logicals){
LncRNA_ASD_mat = bicor(datExpr0[ ,ASD_lncMatch], datExpr0[ ,i], use="p", maxPOutliers = 0.1)
print(sum(i))
lncRNA_ASD_pairs=sum(abs(LncRNA_ASD_mat))

P=10000 ## iterations
sig_pairs=vector(mode = "numeric",length = P)
lnc_num = nrow(LncRNA_ASD_mat)
for (iter in 1:P){
 chosen = sample(1:ncol(datExpr0), lnc_num, replace = FALSE)
 chosen_ASD_mat = bicor( datExpr0[,chosen], datExpr0[ , i], use="p", maxPOutliers = 0.1)
 sig_pairs[iter]= sum(abs(chosen_ASD_mat))
}
Z_scores = scale(c(sig_pairs,lncRNA_ASD_pairs)) ## calculate Z_scores of randomized distribution with actual value on end
Lnc_score = Z_scores[length(Z_scores)] ## Z_score of Actual lncRNA ASD pairs
pnorm(-abs(Lnc_score))

hist(sig_pairs,xlim = range(12000,19000), xlab = "Summed Correlation", main = paste0(titles[j]))
j=j+1
## Use color of line at LncRNAs real value to indicate significance (black = p-value > 0.05 & red = p-value < 0.05)
col="black"
if ( p.adjust(pnorm(-abs(Lnc_score)), n = 2, method = "fdr") <= 0.05 ) {
 col="red" }
abline(v=lncRNA_ASD_pairs, lwd = 3, col = col)

}

## [1] 381

## [1] 490


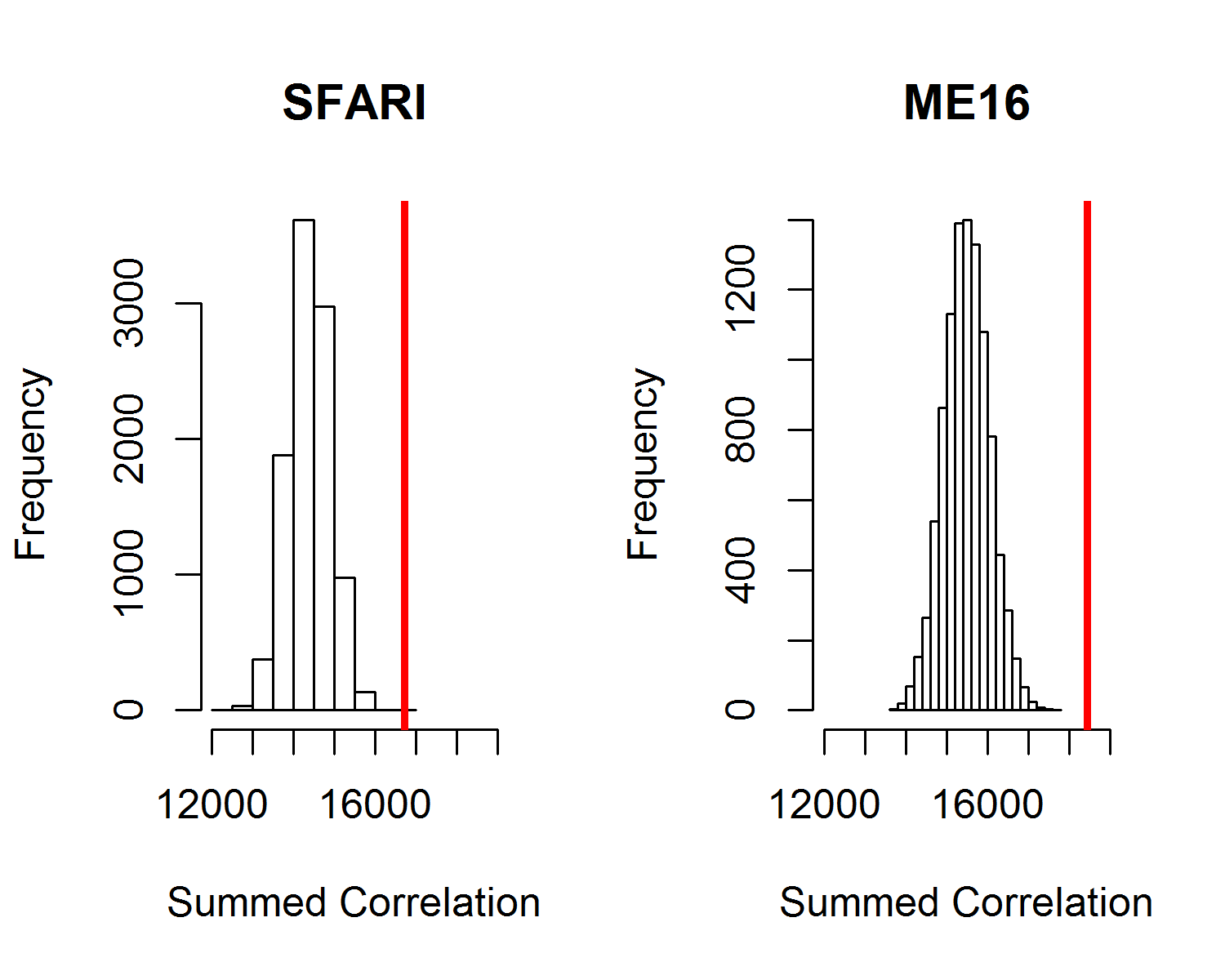


DEG_match = match(rownames(DEG), genelist$ensembl_gene_id[ASD_genes])
DE_ASD = table(is.na(DEG_match))[1]
NotDE_ASD = length(genelist$ensembl_gene_id[ASD_genes]) - DE_ASD
DE_nonASD = nrow(DEG)-length(genelist$ensembl_gene_id[ASD_genes])
NotDE_nonASD = nrow(genelist) - nrow(DEG)

contingency = matrix(nrow=2,ncol=2, data = c(DE_ASD, DE_nonASD, NotDE_ASD, NotDE_nonASD))
rownames(contingency) = c("ASD","non-ASD")
colnames(contingency) = c("DE", "Not DE")
contingency

## DE Not DE
## ASD 47 334
## non-ASD 1221 24586

fisher.test(contingency, "greater")

##
## Fisher's Exact Test for Count Data
##
## data: contingency
## p-value = 4.377e-09
## alternative hypothesis: true odds ratio is not equal to 1
## 95 percent confidence interval:
## 2.030599 3.877353
## sample estimates:
## odds ratio
## 2.833315

GTEx_samples = GTEx_samples[order(GTEx_samples$Tissue), ] ## reorder samples to match GTEx columns (alphabetical)

match_GTEx = match(GTEx$Gene.Name, genelist$gene_symbol)
GTEx$module = genelist$Module[match_GTEx]
GTEx$lncRNA = genelist$lncRNA[match_GTEx]
GTEx$L2FC = genelist$L2FC[match_GTEx]

## remove genes not in network
GTEx = GTEx[!is.na(GTEx$module), ]

exprMat = data.matrix(GTEx[, -c(1,2,56,57,58)])
rownames(exprMat) = GTEx$Gene.Name

##-- filter exprMat to remove tissues with less than 50 samples using GTEx_samples
exprMat = exprMat[GTEx$L2FC !=0 & GTEx$lncRNA , GTEx_samples$Number.of.RNASeq.Samples > 50]

brain_colors = rep("white", ncol(exprMat))
brain_colors[grepl("brain", str_to_lower(colnames(exprMat)))] = "blue"
exprMat = scale(t(exprMat))
exprMat = exprMat[ ,colSums(is.na(exprMat))!=nrow(exprMat)]

my_palette <- colorRampPalette(c("grey","white","orange","orangered","darkred","black"))(n = 599)

heatmap.2(exprMat , dendrogram = "row", trace="none", main="LncRNA Expression by Tissue-Type", notecol="black", key=TRUE, col=my_palette, symm=F, symkey=F, symbreaks=F, key.xlab="Median FPKM Scaled by LncRNA",RowSideColors = brain_colors, margins = c(6,12))


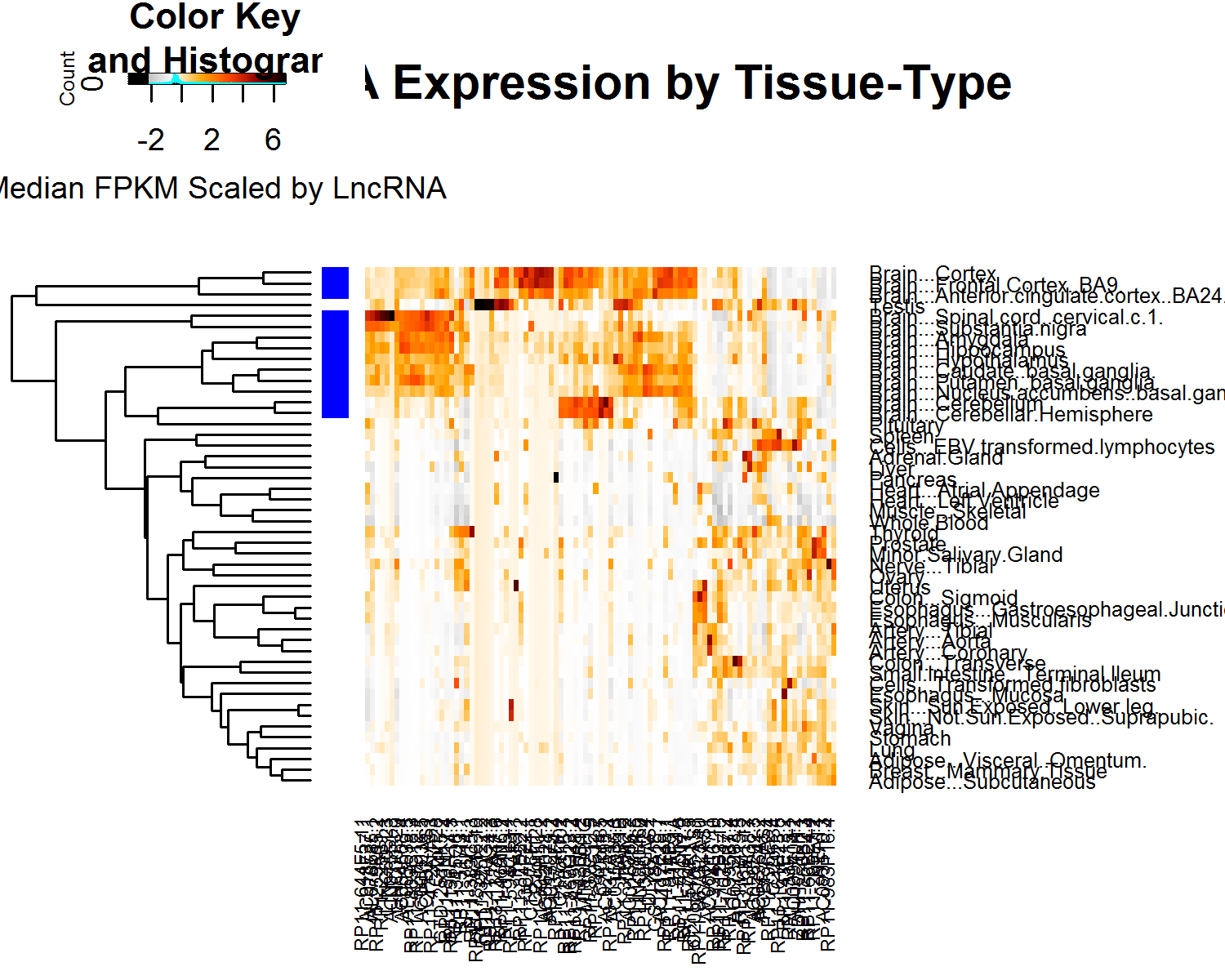


Process all modules for functional enrichments through Gene Ontology Analysis.

library(GOstats)
library(org.Hs.eg.db)

terms_full =data.frame()
for ( mod in unique(moduleColors)){

paramMF <- new("GOHyperGParams", geneIds = genelist$entrez_id[genelist$Module== mod], universeGeneIds = genelist$entrez_id,
 ontology = c("MF"), annotation = "org.Hs.eg", pvalueCutoff = 0.01, testDirection = "over")

paramBP <- new("GOHyperGParams", geneIds = genelist$entrez_id[genelist$Module== mod], universeGeneIds = genelist$entrez_id,
 ontology = c("BP"), annotation = "org.Hs.eg", pvalueCutoff = 0.01, testDirection = "over")
hypMF <- hyperGTest(paramMF)
hypBP <- hyperGTest(paramBP)

termsMF = summary(hypMF)
termsMF$type = "MF"
termsMF$Module = mod
termsMF = rename(termsMF, GO_ID = GOMFID)

termsBP = summary(hypBP)
termsBP$type = "BP"
termsBP$Module = mod
termsBP = rename(termsBP, GO_ID = GOBPID)

terms = rbind(termsMF[1:3, ], termsBP[1:3, ])

terms_full = rbind(terms_full, terms)
}

terms_full = terms_full[rowSums(is.na(terms_full)) != ncol(terms_full), ]
terms_full$Pvalue = p.adjust(terms_full$Pvalue, "fdr") ## correct pvals for # of modules)
write.csv(terms_full, "./Data/GOEnrichmentTable.csv")

Characterize modules enriched for DE lncRNAs by plotting their top Gene Ontology (GO) functional terms and developmental expression profiles.

library(ggplot2)
library(gridExtra)
library(grid)
interesting_mods = c("brown","black","blue") ## all modules enrriched for DE_lncRNAs and/or both ASD gene sets
colnames(MEs0) = stringr::str_sub(colnames(MEs0), start = 3)

for (mod in interesting_mods){

 mod_terms = terms_full[terms_full$Module==mod, ]
 mod_terms = mod_terms[order(mod_terms$Pvalue), ]
 term_names = mod_terms$Term[order(mod_terms$Pvalue)]

 p1 = ggplot(mod_terms, aes( y=-log10(Pvalue), x=reorder(Term, Pvalue), fill= type)) +
 geom_bar( stat = "identity", position = position_dodge()) + theme_bw() +
 #theme(axis.text.x = element_text(angle = 90, hjust = 1)) +
 geom_hline(yintercept = 3, col = "red", lwd=1)+
 coord_flip()+
 ylab(expression(-Log[10]~Pvalue)) + xlab( "GO Term") +ggtitle(paste0(toupper(substr(mod, 1, 1)), tolower(substring(mod, 2))))+
 scale_fill_manual(values=c("#56B4E9", "#E69F00")) +
 theme(plot.margin = unit(c(1,0,1,1), "lines"))

 df = as.data.frame(cbind(Months=clinical$Months.Post.Conception[clinical$Months.Post.Conception <= 22], Expression = MEs0[clinical$Months.Post.Conception <= 22, colnames(MEs0) == mod]) )
 p2= ggplot(df, aes(x = Months, y=Expression))+
 xlab("Months PC")+ ylab("Eigengene (PC1)") +
 geom_point(shape=1, col ="grey") + theme_bw() +geom_jitter(alpha=0.7, shape=1)+
 geom_smooth(method=loess, size=1, col= mod, alpha=.5)+
 geom_vline(xintercept = 10, col="blue", lwd=1)

 assign(paste0(mod,1), p1)
 assign(paste0(mod,2), p2)
}
grid.arrange(blue1, blue2, brown1, brown2, black1, black2, ncol=2, nrow =3, widths =c(2,1), top = textGrob("Module Gene Ontology and Developmental Expression Profile", gp = gpar(fontsize=18)))


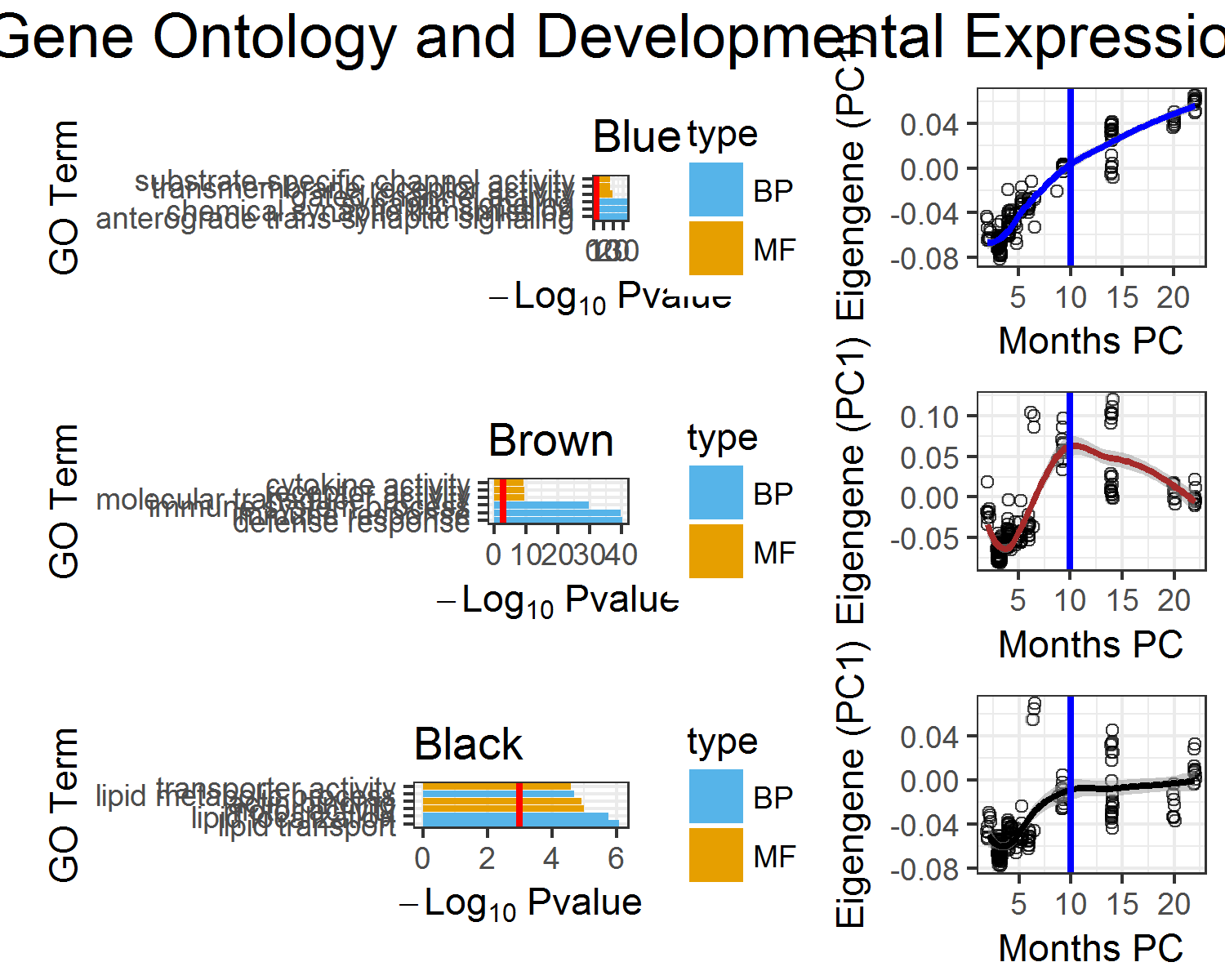


CNV data from SFARI is in cytoband format (ex: 3q29) from different genome assemblies so we will need to create a function to convert all cytoband coordinates into hg38 genomic coordinates.

library(R.utils)
library(stringr)

download.file(url = "http://hgdownload.cse.ucsc.edu/goldenPath/hg38/database/cytoBand.txt.gz", destfile = "cytoBand_HG38.txt.gz" , mode = "wb")
R.utils:::gunzip("cytoBand_HG38.txt.gz", skip=TRUE)

## [1] "cytoBand_HG38.txt"
## attr(,"temporary")
## [1] FALSE

cytobands = read.table(file = "./cytoBand_HG38.txt", sep = "\t")

# Clean up cytobands ------------------------------------------------------
colnames(cytobands) = c("Chromosome","Start","End","Band","Stain")
cytobands$Chromosome = as.character(stringr::str_sub(cytobands$Chromosome, start=4))
### make band entries are written in full with chromosome prefix Ex. 1q43.1
cytobands$Band = paste0(cytobands$Chromosome, cytobands$Band)
cytobands = cytobands[nchar(cytobands$Chromosome) <= 2, ] ## remove alt haplotypes
cytobands = cytobands[cytobands$Chromosome != "M", ] ## remove mitochondria


### CNV_ter checks for "qter"s or "pter"s which indicate entire chromosomal arms affected and outputs the entire range of the affected arm
CNV_ter = function(band, chrom){
 if (grepl("pter", band)) {
 e_pos = max(cytobands$End[cytobands$Chromosome == chrom & grepl(paste0(chrom,"p"),cytobands$Band)])
 s_pos = 0
 }else if (grepl("qter",band)) {
 e_pos = max(cytobands$End[cytobands$Chromosome == chrom & grepl(paste0(chrom, "q"), cytobands$Band)])
 s_pos = min(cytobands$Start[cytobands$Chromosome == chrom & grepl(paste0(chrom, "q"), cytobands$Band)])
}
return(c(s_pos,e_pos))
}

#### this function deals with bands that are too specific for the reference cytobands EX 1q43.11 becomes 1q43.1
### if a match is not found after degeneration; CNV_ter is tried; else return NA
degenerate = function(band, chrom){
 original = band ## make a copy of the input
 while (!(band %in% cytobands$Band) & nchar(band)>1) {
 band = substr(band, 1, nchar(band)-1) ## removes last character from string
 if (substr(band, nchar(band), nchar(band)) == ".") { ## makes sure it does not end on a period
 band = substr(band,1, nchar(band)-1) ## or else it removes another character
 }
 }
 if (band %in% cytobands$Band){
 s_pos = cytobands[cytobands$Band == band, 2]
 e_pos = cytobands[cytobands$Band == band, 3]
 }else if (grepl("pter|qter", original)){ ######## try CNV_ter (check for "q|p ters" else leave NAN)
 ter_check= CNV_ter(original, chrom)
 s_pos=ter_check[1]
 e_pos=ter_check[2]
 }else {
 s_pos= NA
 e_pos = NA
 }
 return( c(s_pos,e_pos))
}

cyto_converter = function(bands, cytobands){
#bands is a list of cytobands for conversion (including chromosome prefix)
#cytobands is a reference dataframe from UCSC with 1st,2nd,3rd,4th columns being Chromosome, Start, End, Band
 bands = as.character(bands)
 starts_vec= vector(mode = "list", length = length(bands))
 ends_vec= vector(mode = "list", length = length(bands))
 chrom_vec= vector(mode = "list", length = length(bands))

 for (i in 1:length(bands)){

 chrom= strsplit(bands[i],"p|q")[[1]][1] #split band on p OR q and select first element
 chrom_vec[i]=chrom
 if (grepl("-",bands[i])) { ##### this chunk deals with joined cytobands. EX. 1q43-1q42
 band_split = strsplit(bands[i],"-")[[1]]
 band_start = band_split[1]
 band_end = paste0(chrom, band_split[2])
 s_pos = cytobands[cytobands$Band == band_start, 2]
 e_pos = cytobands[cytobands$Band == band_end, 3]

 if (!(length(s_pos))) {
 if (sum(grepl(band_start, cytobands$Band[cytobands$Chromosome==chrom]))!=0) {
 matches = cytobands[grepl(band_start, cytobands$Band), ]
 starts_vec[i] = min(matches[, 2])
 } else {
 starts_vec[i]= degenerate(band_start, chrom)[1]
 }
 } else starts_vec[i] = s_pos
 if (!(length(e_pos))) {
 if (sum(grepl(band_end, cytobands$Band[cytobands$Chromosome==chrom]))!=0){
 matches = cytobands[grepl(band_end, cytobands$Band), ]
 ends_vec[i] = max(matches[, 3])
 } else {
 ends_vec[i]= degenerate(band_end, chrom)[2]
 }
 } else ends_vec[i] = e_pos

 }else if (bands[i] %in% cytobands$Band == FALSE ) {
 matches = cytobands[grepl(bands[i], cytobands$Band), ]
 if (nrow(matches) == 0){
 degen_pos = degenerate(bands[i], chrom)
 starts_vec[i] = degen_pos[1]
 ends_vec[i] = degen_pos[2]

 } else if (nrow(matches)!=0){
 starts_vec[i] = min(matches[, 2])
 ends_vec[i] = max(matches[, 3])
 }
 } else {
 s_pos = cytobands[cytobands$Band == bands[i], 2]
 e_pos = cytobands[cytobands$Band == bands[i], 3]
 if (length(e_pos) & (length(s_pos))) {
 starts_vec[i] = s_pos
 ends_vec[i] = e_pos
 }
 }
 }
results_df <- data.frame(bands, unlist(chrom_vec), unlist(starts_vec), unlist(ends_vec))
colnames(results_df)<-c("Cytoband","Chromosome","Start","End")

return(results_df)
}

Now we will use cyto_converter to convert SFARI ASD CNVs into genomic coordinates and then calculate overlaps with the differentially expressed lncRNAs. Finally, we will rank the DE lncRNAs based off of total CNV overlaps and create a prioritized table containing relevant information for the lncRNAs.

library(dplyr)
library(WGCNA)

## ==========================================================================
## *
## * Package WGCNA 1.51 loaded.
## *
## * Important note: It appears that your system supports multi-threading,
## * but it is not enabled within WGCNA in R.
## * To allow multi-threading within WGCNA with all available cores, use
## *
## * allowWGCNAThreads()
## *
## * within R. Use disableWGCNAThreads() to disable threading if necessary.
## * Alternatively, set the following environment variable on your system:
## *
## * ALLOW_WGCNA_THREADS=<number_of_processors>
## *
## * for example
## *
## * ALLOW_WGCNA_THREADS=4
## *
## * To set the environment variable in linux bash shell, type
## *
## * export ALLOW_WGCNA_THREADS=4
## *
## * before running R. Other operating systems or shells will
## * have a similar command to achieve the same aim.
## *
## ==========================================================================

CNV_df = cyto_converter(CNVs$CNV.Locus, cytobands = cytobands)
table(rowSums(is.na(CNV_df)))

##
## 0 1 2
## 5070 2 2

CNV_df = CNV_df[rowSums(is.na(CNV_df))==0, ]


CNVs = makeGRangesFromDataFrame(CNV_df)

genelist_Gr = makeGRangesFromDataFrame(genelist[!is.na(genelist$start), colnames(genelist)=="chromosome" | colnames(genelist)=="start" | colnames(genelist)=="end" ], start.field = "start", end.field = "end" )
names(genelist_Gr) = genelist$gene_symbol[!is.na(genelist$start) ]

overlaps = countOverlaps( genelist_Gr, CNVs)
genelist$overlaps = overlaps[match(genelist$gene_symbol, names(overlaps))]

ranked = genelist[genelist$lncRNA == TRUE & genelist$L2FC !=0, ]
ranked = select(ranked, -c(row_num, gene_id, entrez_id, chromosome, start,end, ASD_score, lncRNA, ME16))

mod_BPs = terms_full %>%
 filter(type=="BP") %>%
 group_by(Module) %>%
 filter( Pvalue==min(Pvalue) & !duplicated(Pvalue))

ranked$Module_BP = mod_BPs$Term[match(ranked$Module, mod_BPs$Module)]

top_cors = data.frame()
for ( i in 1:nrow(ranked)){
 lncRNA = ranked$ensembl_gene_id[i]

 lnc_expr = datExpr0[ ,colnames(datExpr0)== lncRNA]

 cor_mat = bicor(datExpr0[ , genelist$biotype=="protein_coding"], lnc_expr, use="p", maxPOutliers = 0.1)
 #which.max(cor_mat)
 ind = cor_mat[which.max(cor_mat), ]

 gene_name = genelist[genelist$ensembl_gene_id == names(ind), ]
 # near_cor = bicor(datExpr0[ ,colnames(datExpr0) == E_ID], lnc_expr, use="p", maxPOutliers = 0.1)
 # gene_name$nearest_cor = near_cor

 gene_name$Top_cor = as.numeric(ind)
 top_cors = rbind(top_cors, gene_name)
}
top_cors = select(top_cors, -c(row_num, gene_id, ensembl_gene_id, entrez_id, chromosome, start, end, lncRNA, ME16))
ranked = cbind(ranked,top_cors)

ranked = ranked[order(ranked$overlaps, decreasing = TRUE), ]
head(ranked)

## ensembl_gene_id gene_symbol L2FC biotype
## 50258 ENSG00000257151 RP11-701H24.2 -1.112526 lincRNA
## 17064 ENSG00000188511 C22orf34 1.310380 lincRNA
## 30465 ENSG00000228459 CXorf28 3.261527 lincRNA
## 21008 ENSG00000205634 RP11-191L9.6 -2.773085 lincRNA
## 27300 ENSG00000224271 RP11-191L9.4 -2.049563 lincRNA
## 32915 ENSG00000231711 RP11-398F12.1 1.520879 processed_transcript
## DEG_lncRNA_nearest_gene nearest_ASD_score Module membership overlaps
## 50258 SNORD116-1 <NA> purple 0.8600899 84
## 17064 BRD1 <NA> brown 0.5737664 64
## 30465 MXRA5 <NA> brown 0.6022096 30
## 21008 TBC1D22A <NA> brown 0.1939161 28
## 27300 TBC1D22A <NA> brown 0.2133065 28
## 32915 C22orf26 <NA> green 0.7248878 28
## Module_BP
## 50258 positive regulation of phosphoprotein phosphatase activity
## 17064 defense response
## 30465 defense response
## 21008 defense response
## 27300 defense response
## 32915 drug catabolic process
## gene_symbol L2FC biotype ASD_score
## 50258 ATRNL1 -1.447133 protein_coding 5
## 17064 NOTCH4 0.000000 protein_coding 0
## 30465 CHAD 0.000000 protein_coding 0
## 21008 FBXO40 -1.434994 protein_coding 4
## 27300 SMYD1 0.000000 protein_coding 0
## 32915 ACACB 0.000000 protein_coding 0
## DEG_lncRNA_nearest_gene nearest_ASD_score Module membership overlaps
## 50258 <NA> <NA> purple 0.9334695 12
## 17064 <NA> <NA> brown 0.7773115 5
## 30465 <NA> <NA> brown 0.9029223 6
## 21008 <NA> <NA> blue 0.8078825 11
## 27300 <NA> <NA> blue 0.8471307 7
## 32915 <NA> <NA> brown 0.8230040 5
## Top_cor
## 50258 0.8750336
## 17064 0.6580880
## 30465 0.6924504
## 21008 0.9169690
## 27300 0.8395817
## 32915 0.8126487

write.csv(ranked, "./Data/ranked_lncRNAs.csv", row.names = FALSE)

## lncRNA genomic and coexpression characterization

lncRNAs %>%
 group_by(Module) %>%
 filter(n() > 3) %>%
 ggplot( aes(factor(1), fill = Module)) +
 theme_bw() +
 geom_bar(width =1) +
 scale_x_discrete(NULL, expand = c(0, 0)) +
 scale_y_continuous(NULL, expand = c(0, 0))+
 coord_polar(theta="y") +
 scale_fill_manual(values = c("black","blue", "brown","green","grey","turquoise"))


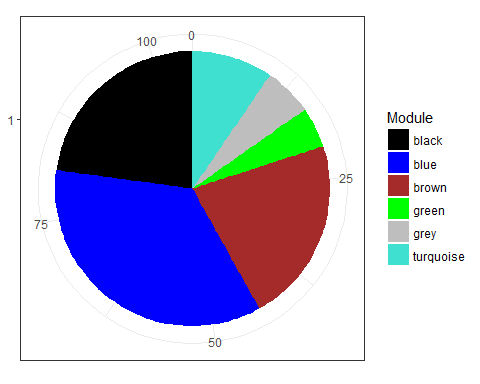


# Tissue_specificity ------------------------------------------------------

# re-load GTEx data

GTEx = read.csv("./Data/RAW/GTEx/GTEx_Analysis_v6_RNA-seq_RNA-SeQCv1.1.8_gene_median_rpkm.csv")

GTEx_samples = read.csv("./Data/RAW/GTEx//GTEx_sample_metadata.csv")

Ensembls = unlist(lapply(strsplit(as.character(GTEx$Ensembl_gene_ID),"\\."), "[[",1))
rownames(GTEx) = Ensembls
GTEx = GTEx[ ,-c(1,2)]

GTEx_samples = GTEx_samples[order(GTEx_samples$Tissue), ] ## reorder samples to match GTEx columns (alphabetical)
GTEx = GTEx[ , GTEx_samples$Number.of.RNASeq.Samples > 50]


match_GTEx = match(rownames(GTEx), lncRNAs$ensembl_gene_id)

GTEx = GTEx[!is.na(match_GTEx), ]

library(stringr)
brain_colors = rep("white", ncol(GTEx))
brain_colors[grepl("brain", str_to_lower(colnames(GTEx)))] = "blue"

brain_ratios = vector(mode = "numeric", length = nrow(GTEx))
for ( i in 1:nrow(GTEx)){
 brain_ratios[i] = sum(GTEx[i, brain_colors=="blue"])/sum(GTEx[i,]) * 100
}

lncRNAs$Brain_ratio = brain_ratios[match(lncRNAs$ensembl_gene_id, rownames(GTEx))]


lncRNAs %>%
 group_by(Module) %>%
 filter(n() > 3) %>%
 summarise(mean_brain = mean(Brain_ratio, na.rm =TRUE)) %>%
 ggplot() +
 geom_bar(mapping = aes(x=Module,y=mean_brain, fill = Module), stat = "identity") +
 scale_fill_manual(values = c("black","blue", "brown","green","grey","turquoise"))+
 theme_bw()+
 geom_hline(yintercept = 50, col="red")


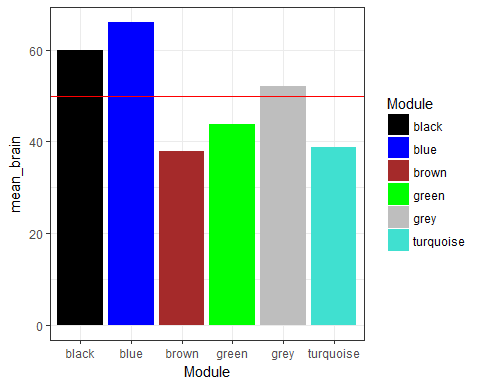


lncRNAs %>%
 group_by(Module) %>%
 filter(n() > 3) %>%
 summarise(CNV_overlaps = sum(overlaps, na.rm =TRUE)) %>%
 ggplot() +
 geom_bar(mapping = aes(x=Module,y = CNV_overlaps, fill = Module), stat = "identity") +
 scale_fill_manual(values = c("black","blue", "brown","green","grey","turquoise"))+
 theme_bw()


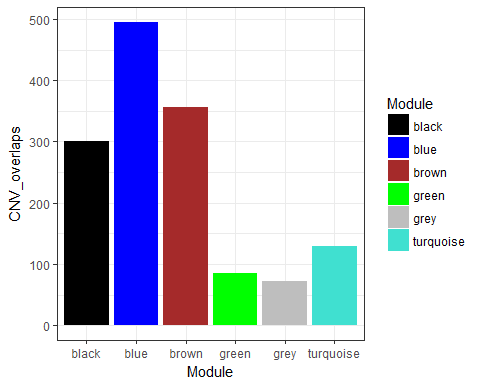

Supplement: S2 Text — (DOCX) [file pone.0178532.s005.docx]
